# Histone and non-histone protein acetylation in a tail regeneration model (*Ambystoma mexicanum*)

**DOI:** 10.1242/bio.062726

**Published:** 2026-07-14

**Authors:** Nataliya Timoshevskaya, Raissa F. Cecil, James Schwartz, Jeramiah J. Smith, S. Randal Voss

**Affiliations:** ^1^ Department of Neuroscience, Spinal Cord and Brain Injury Research Center, and Ambystoma Genetic Stock Center, University of Kentucky, Lexington, KY 40536, USA; ^2^ Department of Biology, University of Kentucky, Lexington, KY 40506, USA

**Keywords:** Axolotl, Regeneration, Transcription, HDAC, HDACi, Acetylation, Non-histone protein

## Abstract

Histone deacetylase inhibitors (HDACi) potently inhibit appendage regeneration, but how HDACi alter protein acetylation is relatively unexplored. Using genomic, transcriptomic, and proteomic approaches, we report HDACi-mediated changes in gene expression and protein acetylation at the outset of *Ambystoma mexicanum* tail regeneration. HDACi (romidepsin) treatment globally reversed a genome-wide deacetylation response after tail amputation, broadly increasing H3K9ac and H3K27ac within intergenic regions, gene promoters, and gene bodies. Changes in promoter acetylation were only weakly associated with changes in gene expression when considering all acetylated genes. However, the magnitude of acetylation change was significantly greater for upregulated genes, including previously identified regeneration-inhibitory genes. We further detected hundreds of differentially acetylated non-histone proteins after tail amputation, including proteins that function in transcriptional regulation and hemostasis. We used a small molecule (A-485) to inhibit differentially acetylated transcription factors (Crebbp/Ep300) to show their requirement for tail regeneration. A-485 downregulated many of the same genes that were downregulated by romidepsin, but the upregulated gene set was relatively unique. Our results implicated histone and non-histone protein acetylation in the early transcriptional regulation of regeneration associated genes.

## Teaser

**Summary:** HDAC inhibitor blockade of appendage regeneration is associated with genome-wide histone hyperacetylation, increased promoter acetylation and transcription of inhibitory genes, and differential acetylation of transcription factors that likely regulate regeneration-permissive genes.

## INTRODUCTION

Histone deacetylase (HDAC) protein activity is required for successful tissue regeneration in a variety of organisms, including amphibians that serve as primary models in studies of vertebrate appendage regeneration (Tseng et al., 2011; Taylor and Beck, 2012; Voss et al., 2019, 2021; Wang et al., 2019; Arbach et al., 2022). Studies have shown that transcription is altered when HDAC inhibitors are applied to regenerating tissues; however, very little is known about the underlying molecular mechanisms. HDACs enzymatically remove acetyl from the Nε-amino group of lysine residues in histones and non-histone proteins (Taunton et al., 1996). Histone deacetylation is generally associated with chromatin compaction and transcriptional repression, as the removal of negatively charged acetyl-lysine residues from histone tails increases the affinity of DNA binding to nucleosomes, creating a relatively inaccessible chromatin state for RNA Pol II-mediated transcription (Marmorstein and Roth, 2001; Eberharter and Becker, 2002). HDAC inhibitors (HDACi) block histone deacetylation, thus enabling lysine acetyltransferases to create hyperacetylated, accessible chromatin states that are associated with transcriptional activation. However, HDACi not only increase transcription, they also decrease transcription (Chou et al., 2011; Greer et al., 2015). Moreover, HDACi simultaneously and reproducibly increase and decrease the transcription of gene sets with correlated functions, supporting the idea that acetylation balance is critical for regulating biological processes (Chen et al., 1999; Voss et al., 2021). For example, HDACi have been shown to upregulate stress, growth arrest, and apoptotic genes either directly through histone hyperacetylation and the generation of accessible chromatin (Marks et al., 2000), or indirectly by inducing reactive oxidative injury (Rosato and Grant, 2005; Baddar et al., 2019), or by altering the activities of cell signaling proteins and transcription factors (Gu and Roeder, 1997; Choudhary et al., 2009). Moreover, histone hyperacetylation, which is generally thought to characterize transcriptionally accessible chromatin, may increase the writing of transcriptionally repressive post-translational modifications (PTMs) and recruit transcriptionally repressive proteins (Halsall et al., 2015; Agricola et al., 2011). Accordingly, transcriptional regulation and output may not simply be associated with chromatin-level changes in histone acetylation.

In comparison to the well-characterized effects of HDAC on histone deacetylation, less is known about lysine deacetylation of non-histone proteins (Narita et al., 2019). The first non-histone protein to be identified as a target for lysine acetylation was Tp53 (Gu and Roeder, 1997). Although Tp53 transcriptional activity is modified by several PTMs, acetylation is indispensable for its tumor suppressor function (Tang et al., 2008; Yamaguchi et al., 2009). Site-specific acetylation affects Tp53 stability and provides a code for recruiting proteins that mediate different transcriptional outcomes at target genes and enact diverse biological processes including cell differentiation, proliferation, growth, and apoptosis (Xia et al., 2022). Acetylation also increases the activity of other key transcription factors, including Hif1α, Stat3, Gata1, Runx1, and Ep300/Crebbp (Boyes et al., 1998; Thompson et al., 2004; Yuan et al., 2005; Wang et al., 2009a; Geng et al., 2012). While Ep300/Crebbp are perhaps best known for acetylating H3K27 and activating transcription through chromatin remodeling, these paralogs are predicted to acetylate over 5000 protein targets (Weinert et al., 2018). Thus, mechanisms of histone and non-histone acetylation likely overlap in regulating biological processes (Smith, 2008).

Our previous studies of *Ambystoma mexicanum* (axolotl) tail regeneration revealed several insights about HDAC inhibitors that mirror findings from other regeneration studies as well as studies of cancer cells. First, HDACi have rapid and dynamic effects on biological processes. For example, we previously showed that a 1-min post-amputation treatment of romidepsin, a specific Class I HDACi, blocked axolotl tail regeneration (Voss et al., 2019). Thus, transcriptional programs that are immediately triggered in response to amputation injury require HDAC activity. Regeneration-inhibitory concentrations of romidepsin only appear to affect cells in the injury environment. Thus, the effects of romidepsin are context dependent, specifically altering transcription of injury responsive cells. As romidepsin was initially US Food and Drug Administration (FDA) approved for T-cell lymphoma (Grant et al., 2010), this suggests that injury-responsive cells in axolotls may share molecular properties of transcriptional regulation with transformed cancer cells. We also found, as has been shown in cancer studies (e.g. Hamam and Palaniyar, 2019), that HDACi function in a concentration-dependent manner to affect transcription, suggesting that a proper balance of acetylation is essential for transcriptional regulation (Voss et al., 2021). Above a threshold level, romidepsin inhibited regeneration and increased the expression of genes that function in inflammation, negative regulation of cell signaling, and growth arrest. We collectively refer to such genes as regeneration inhibitory. In contrast and again above a threshold level, romidepsin decreased the expression of genes that are typically upregulated during normal regeneration. We collectively refer to such genes as regeneration permissive because they support progenitor cell activation, proliferation, and subsequent tissue patterning.

**Fig. 1.**
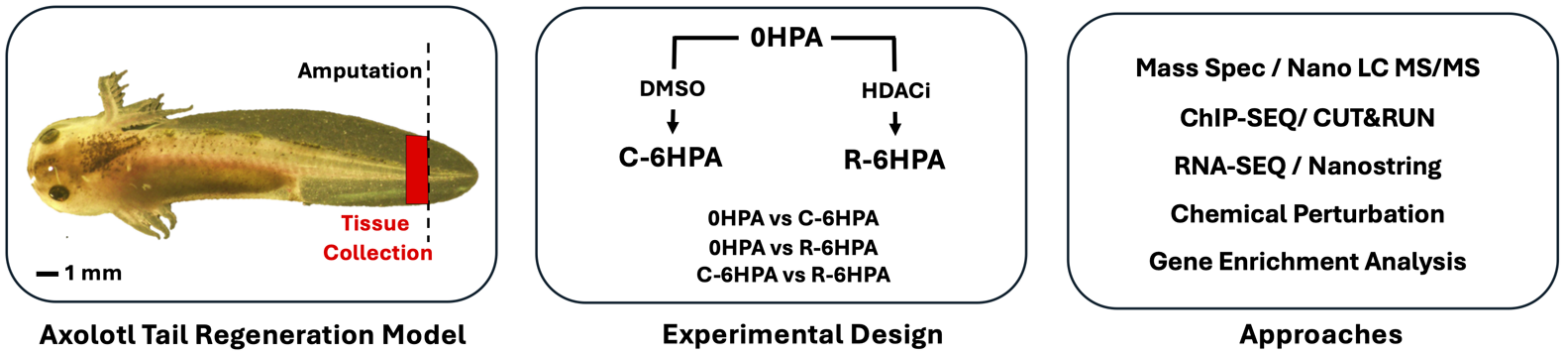
**Overview of the regeneration model, experimental design, and approaches used in the study.** Axolotl embryos were administered tail amputations and treated with DMSO (C) versus romidepsin (R) to detail early [6 h post amputation (6HPA)] protein acetylation changes using proteomic, epigenetic, and transcriptomic approaches.

Few studies have characterized HDACi-mediated effects on histone and non-histone protein acetylation during tissue regeneration – changes that hypothetically may associate with transcriptional regulation and regeneration outcome. Here, we build upon previous transcriptional studies of romidepsin to examine protein acetylation changes during axolotl tail regeneration (Ponomareva et al., 2015) (Fig. 1). We collected tissue samples at early post-injury timepoints to capture both wound-healing and early regeneration responses. Although acetylation studies typically use cell lines to control for cell-cell variability, we previously showed using single-nucleus RNA sequencing (snRNA-SEQ) that romidepsin alters the expression of key regeneration genes in the same direction across diverse cell types of the axolotl tail (Voss et al., 2021). This suggests that HDAC activity may act to coordinate similar transcriptional responses across cells after injury. Using genomic, transcriptomic, and proteomic approaches, we detail dramatic changes in histone acetylation, gene expression and protein acetylation at the outset of tail regeneration. Our results suggest a role for histone and non-histone acetylation in regulating gene expression during tissue regeneration.

## RESULTS

### Romidepsin-associated changes in acetylation across histone types

**Fig. 2.**
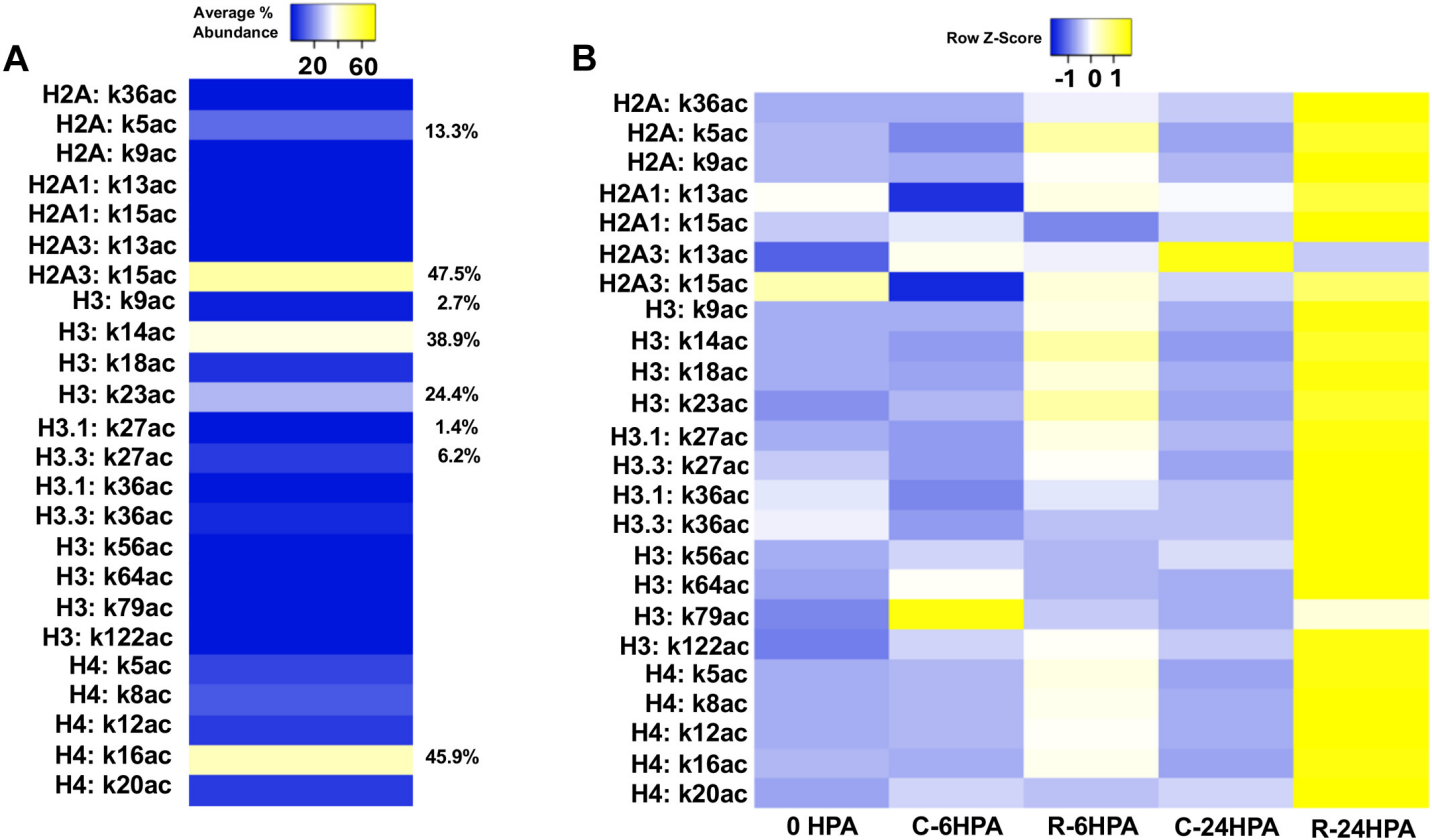
**Results of mass spectrometry screen of histone acetylation PTMs for axolotl embryo tail regeneration.** (A) Heatmap showing percent average abundance of each acetylated PTM (relative to unacetylated PTM) across samples (0-HPA, C-6HPA, R-6HPA, C-24HPA, R-24HPA). (B) Heatmap showing Z-scores for each PTM across samples.

Multiple lysine residues within histones are accessible for acetylation and deacetylation. To broadly screen for acetylation changes across a representative sample of histone types and subtypes, we quantified the relative abundance of lysine acetylation modifications using protein mass spectrometry. Developmental stage 42 axolotl embryos (Ponomareva et al., 2015) were administered tail amputations, and tissues at the distal tip were collected at the time of amputation (0-HPA; HPA, h post amputation) and 6 and 24 h later, after treatment in either 0.01% DMSO (C-6HPA, C-24HPA) or a tail regeneration-inhibitory dose of 10 μM romidepsin (R-6HPA, R-24HPA). The average abundance of acetylated lysine residues across all five samples, in comparison to non-acetylated lysine residues, was generally low and variable among the sites that were surveyed (Fig. 2A). Less than 10% of lysine residues were acetylated for 19 of the 24 sites surveyed. The sites with the highest levels of acetylation included H2A3K15 (47.5%), H4K16 (45.9%), H3K14 (38.9%), and H3K23 (24.4%). While very little is known about the function of H2A3K15ac, the other histone modifications have been associated with transcriptional activation (Ma et al., 2016; Pal et al., 2023; Regadas et al., 2021). In comparison to these relatively highly acetylated sites, canonical markers of transcriptional activation presented much lower levels of acetylation (e.g. H3K9ac=2.7%; H3.1K27ac=1.4%, H3.3K27ac=6.2%); presumably this reflects the relatively small contribution of promoter and enhancer sequences to overall genome size. However, even though sites presented considerable variation in lysine acetylation abundance, a majority showed increased acetylation in response to romidepsin treatment (Fig. 2B). At 6HPA, acetylation was higher in romidepsin- versus DMSO-treated embryos for 18 of 24 sites, and at 24HPA, 23 of 24 sites showed higher levels of acetylation. Overall, romidepsin increased acetylation across multiple histone types, a pattern consistent with hyperacetylation.

### Genome-level effects of romidepsin on H3K9ac and H3K27ac

**Fig. 3.**
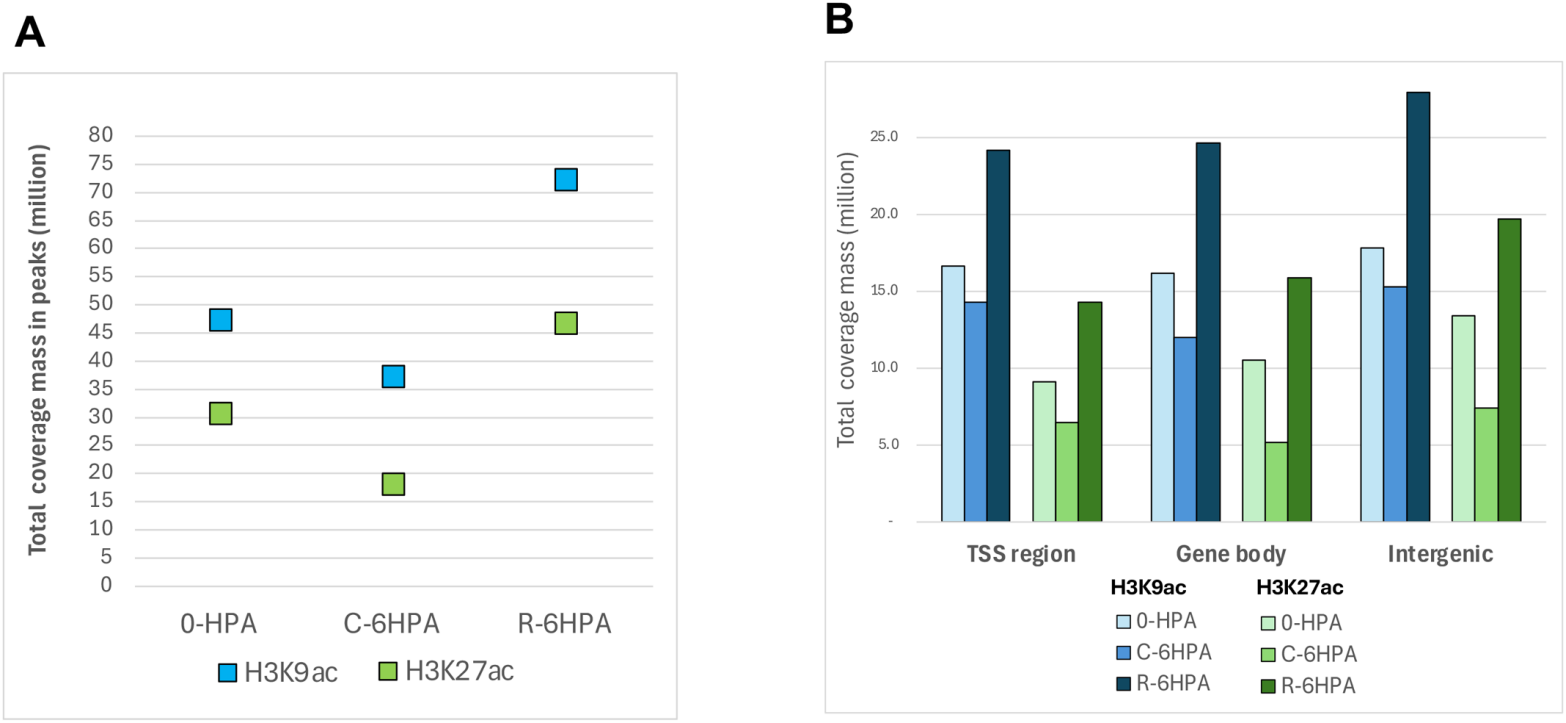
**Genomic patterns of H3K9ac and H3K27ac coverage at the outset of axolotl embryo tail regeneration.** (A) Total mass of ChIP-SEQ coverage across the genome for 0-HPA, C-6HPA, and R-6HPA samples. (B) Total mass of ChIP-SEQ coverage for different genomic features, including promoters, gene bodies, and intergenic regions.

We next performed chromatin immunoprecipitation sequencing (ChIP-SEQ) to investigate genome-wide histone acetylation dynamics. Using the tail amputation model described above, we collected distal tail tip pieces for the following time points: 0-HPA, C-6HPA, and R-6HPA. For each of these samples, we performed ChIP-SEQ using antibodies that bind H3K9ac and H3K27ac residues, and isolated and sequenced associated DNA to generate >90 million single-end sequence reads for each sample. After mapping and filtering, the number of retained reads per sample varied between 11 and 30 million, with more reads retained for 0-HPA and R-6HPA than for C-6HPA pulldowns (Table S1). Narrow peaks for each sample were identified using model-based analysis of ChIP-SEQ (MACS2) (Feng et al., 2012) and demonstrated sufficient Frip-score values (H3K9ac: 0-HPA 0.224, C-6HPA 0.127, R-6HPA 0.331; H3K27ac: 0-HPA 0.133, C-6HPA 0.045, R-6HPA 0.22). To facilitate comparison of acetylation between samples, we combined peaks from all three samples to define a union set of peak intervals for H3K9ac and separately for H3K27ac. We observed greater overall peak mass (the sum of normalized coverage values over peak intervals) for R-6HPA samples than C-6HPA samples for both PTMs. On average, union peak mass decreased at C-6HPA compared to 0-HPA, indicating a widespread decrease in H3K9ac and H3K27ac at the outset of normal regeneration. In contrast, acetylation of these normally deacetylated sites was much greater for the R-6HPA sample (Fig. 3A; Table S1). To validate the ChIP-SEQ data, CUT&RUN was performed using the H3K27ac antibody; the resulting data again showed greater peak mass for the R-6HPA sample (Fig. S1A). Observation of increased histone acetylation in R-6HPA versus C-6HPA samples, for independent H3K9ac and H3K27ac PTMs and corroborated by two different methodologies, extended the mass spectrometry results above by showing a genome-wide increase in histone acetylation.

### Distribution of H3K9ac and H3K27ac peaks across genomic features

We next investigated the distribution of H3K9ac and H3K27ac PTMs among three different compartments of the axolotl genome: promoters [2 kb upstream and downstream of the transcription start site (TSS)], gene bodies (from 2 kb downstream of the TSS to the transcription end site), and intergenic regions. Patterns of acetylation discovered in the genome-wide analyses above were observed for all three compartments (Fig. 3B). In the CUT&RUN experiment, the acetylation mass was also much higher in the R-6HPA sample for promoters, gene bodies, and intergenic regions, consistent with global hyperacetylation (Fig. S1B). To investigate acetylation in promoters more closely, we generated coverage profiles across 19,057 non-overlapping genes (size>8 kb). Overall, the R-6HPA profile was highest for both PTMs. Moreover, acetylation peaks were broader for R-6HPA with evidence of acetylation spreading from promoters into gene bodies (Fig. S2).

**Fig. 4.**
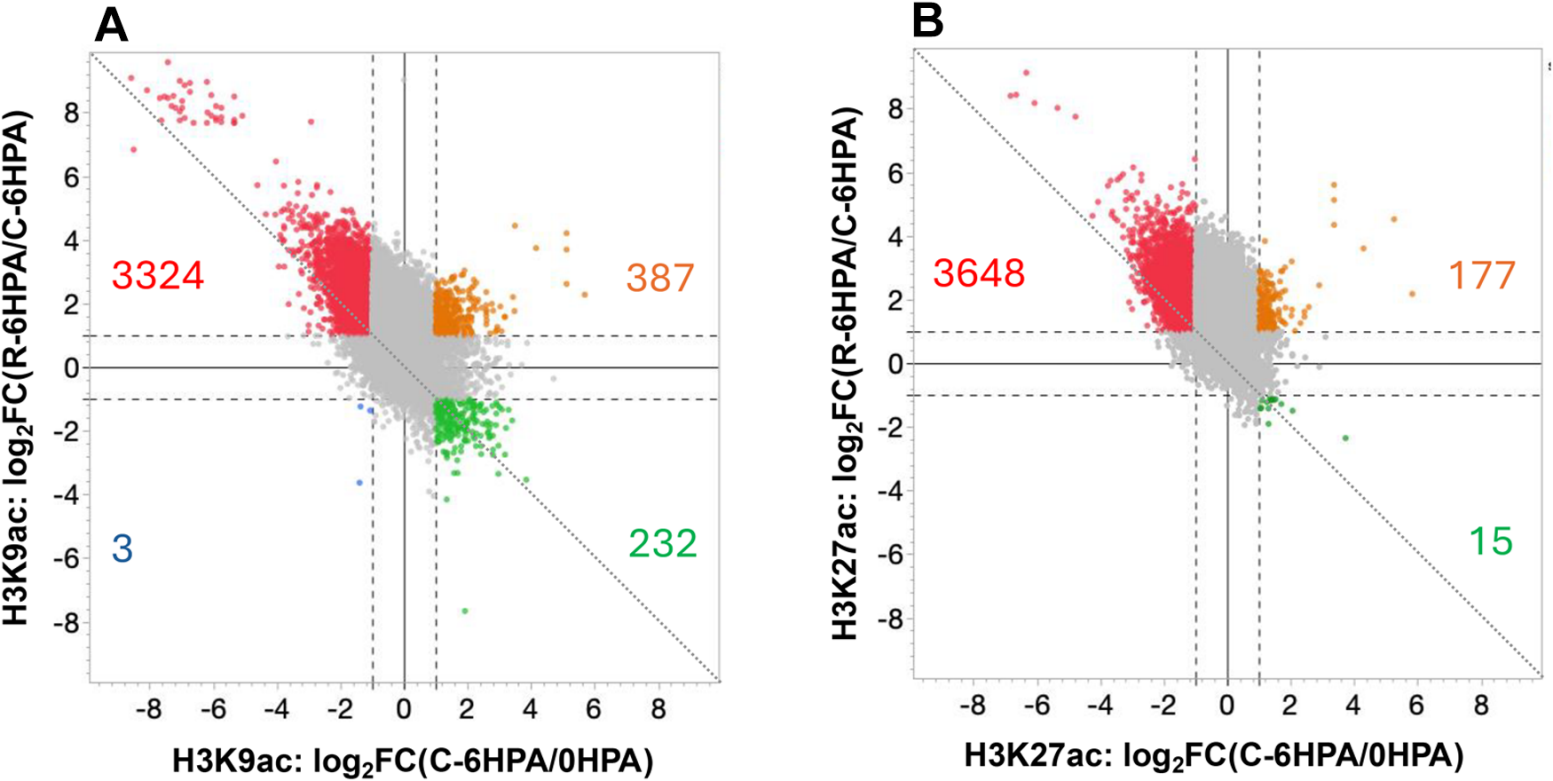
**Promoter acetylation changes after amputation injury.** (A) Changes in H3K9ac for 16558 promoters with coverage mass >200 in at least one sample. (B) Changes in H3K27ac for 13,733 promoters with coverage mass >200 in at least one sample. Colored dots: promoters that have ≥twofold difference for both comparisons. Red dots: promoters that show decreased acetylation under control conditions are increased by romidepsin treatment. Orange dots: promoters that show increased acetylation under control conditions are increased further by romidepsin treatment. Green dots: promoters that show increased acetylation under control conditions are decreased by romidepsin treatment. Blue dots: promoters that show decreased acetylation under control conditions are decreased further by romidepsin treatment.

Several thousand promoters showed at least a twofold depletion of H3K9ac (*N*=3902) and H3K27ac (*N*=3870) marks between C-0HPA and C-6HPA samples, whereas a smaller fraction showed a twofold increase in acetylation (H3K9ac: *N*=1165; H3K27ac: *N*=327). Most promoters that showed depletion of acetyl marks following tail amputation retained acetylation under romidepsin treatment (H3K9ac: 85%, H3K27ac: 94%), with acetylation levels generally greater than or similar to baseline (C-0HPA) (Fig. 4). The relatively fewer number of promoters that showed increased acetylation after tail amputation were variably affected by romidepsin. For H3K9ac, 33% of promoters were further increased by twofold, and 19% had a twofold decrease; for H3K27ac, 54% of promoters were further increased by twofold and only 5% were decreased by twofold. Thus, almost all genes that showed depletion of H3K9ac and H3K27ac after amputation injury, and more than half of genes with increased acetylation of H3K27ac were susceptible to romidepsin-mediated acetylation.

### Relationship between histone acetylation and gene expression

**Fig. 5.**
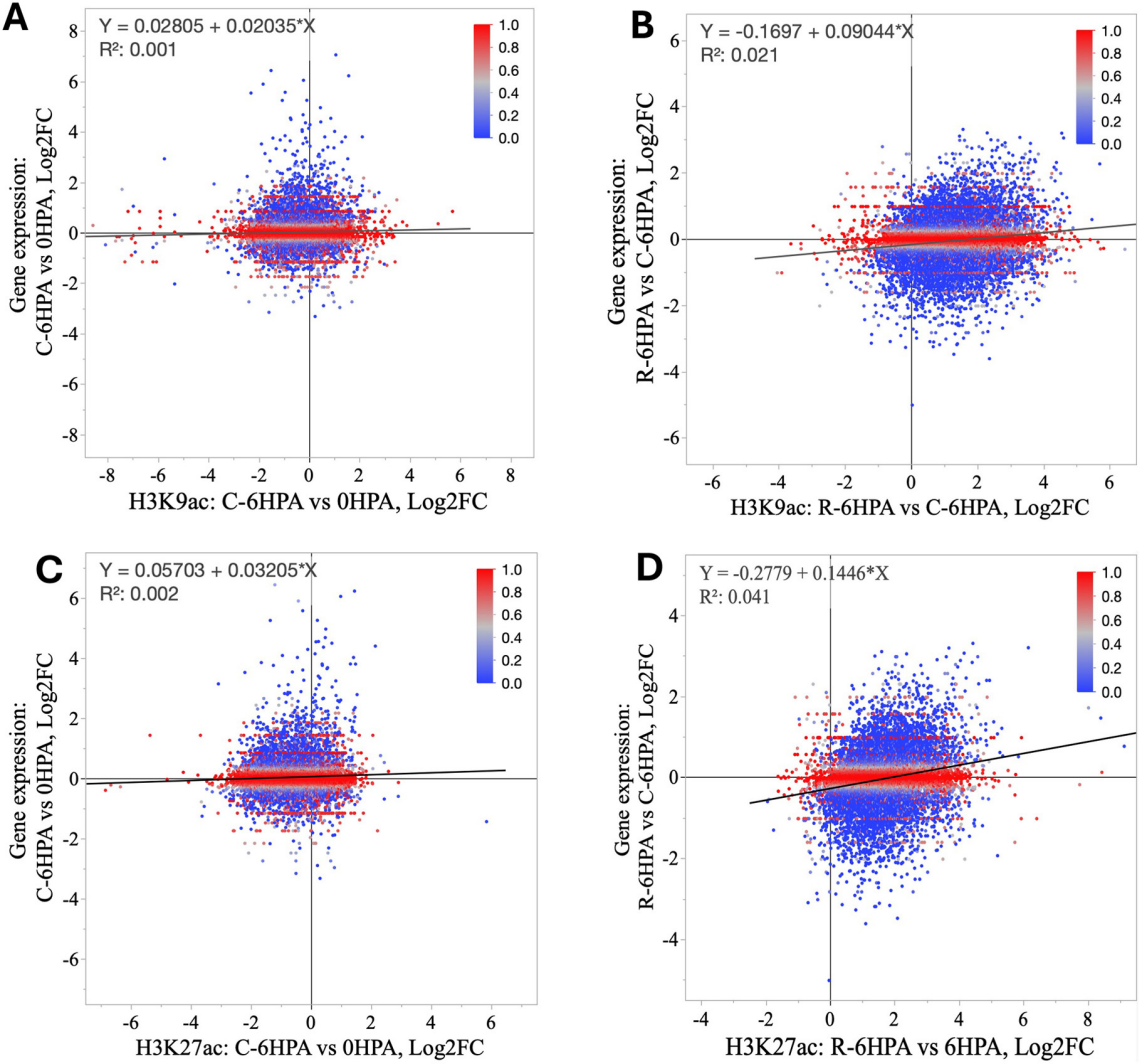
**Comparison of promoter acetylation and gene expression changes.** (A) Log_2_ gene expression change between 0-HPA and C-6HPA samples for 13,598 genes with H3K9ac promoter mass >200 in at least one sample. (B) Log_2_ gene expression change between C-6HPA and R-6HPA samples for 13,598 genes with H3K9ac promoter mass >200 in at least one sample. (C) Log_2_ gene expression change between 0-HPA and C-6HPA samples for 11,298 genes with H3K27ac promoter mass >200 in at least one sample. (D) Log_2_ gene expression change between C-6HPA and R-6HPA samples for 11,298 genes with H3K27ac promoter mass >200 in at least one sample. Dots are colored to show the *P*-value for differential gene expression.

**Fig. 6.**
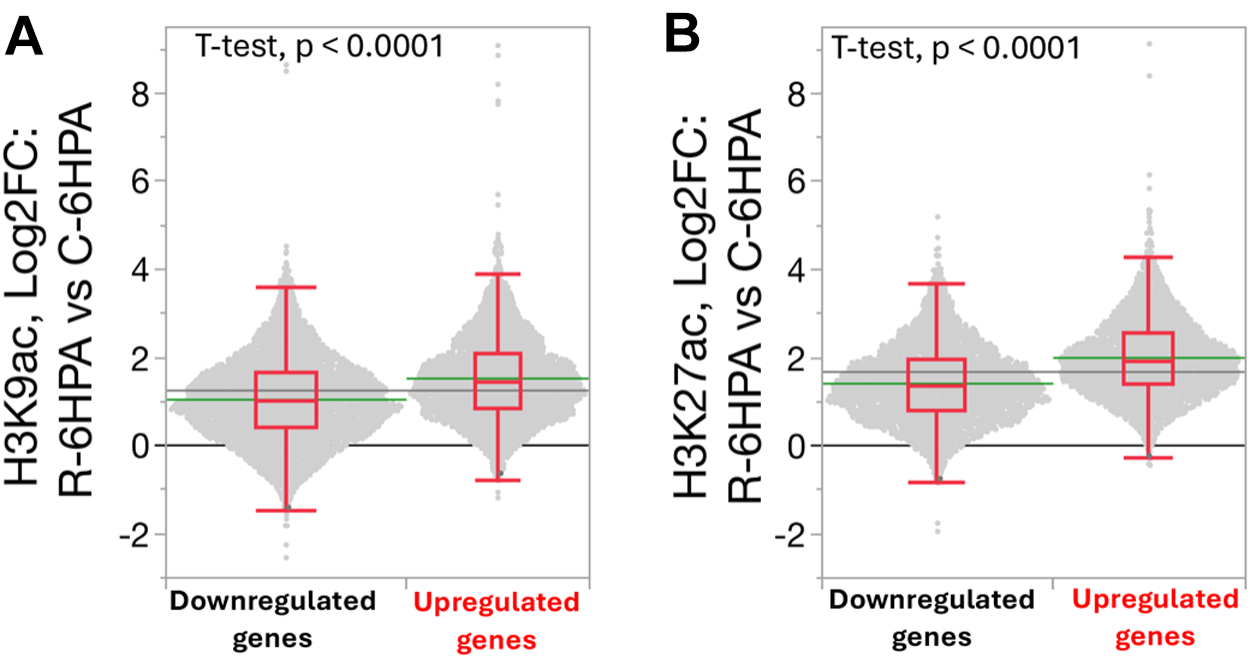
**Magnitude of promoter acetylation change identified between C-6HPA and R-6HPA samples for upregulated and downregulated genes (*P*<0.1) with evidence of acetylation.** (A) Log2 H3K9ac change for 2187 upregulated and 2873 downregulated genes. (B) Log_2_ H3K27ac change for 1937 upregulated and 2327 downregulated genes. Green lines indicate mean values for each category. *P*-values are based on a two-tailed *t*-test assuming unequal variances.

**Fig. 7.**
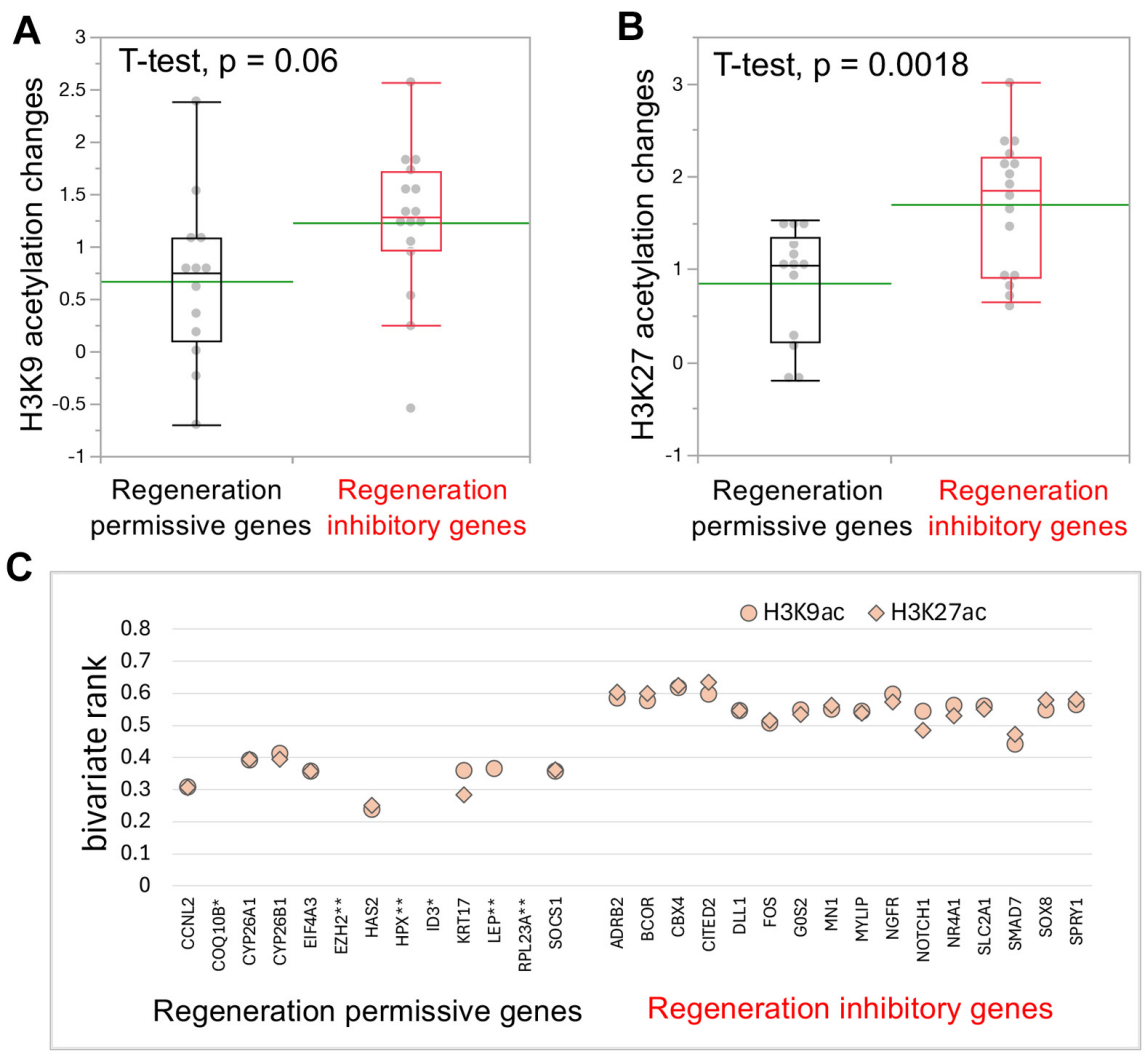
**Magnitude of promoter acetylation change for 29 regeneration-permissive and -inhibitory genes between C-6HPA and R-6HPA samples.** (A,B) H3K9ac (A) and H3K27ac (B) *P*-values are based on a two-tailed *t*-test assuming unequal variances. (C) Bivariate (acetylation and gene expression) rank order of regeneration-permissive and -inhibitory genes.

Many studies have shown that changes in histone acetylation are associated with changes in transcription. To assess this, we examined the relationship between promoter acetylation and gene expression using snRNA-SEQ data from a previous study of 0-HPA, C-6HPA, and R-6HPA samples (Voss et al., 2021). When considering all expressed genes with evidence of acetylation (promoter mass>200 in at least one sample), the association between acetylation change and gene expression change was positive but extremely weak for the comparison of control samples (0-HPA versus C-6HPA) (Fig. 5A,C), and only slightly stronger when comparing C-6HPA and R-6HPA samples (Fig. 5B,D). These trends were observed for both H3K9ac and H3K27ac. Considering that romidepsin globally increased promoter acetylation across many genes, the relatively weak association suggested a more nuanced effect on gene expression. To investigate this further, we statistically evaluated the magnitude of increase in promoter acetylation for genes that were differentially expressed with *P*-value<0.1 between C-6HPA and R-6HPA. We found that the magnitude was significantly higher for genes that were upregulated versus downregulated by romidepsin (Fig. 6A,B); again, these results were observed for both H3K9ac and H3K27ac. To determine if these genes share functional properties, we performed gene enrichment analyses using genes with the highest (top 5%) bivariate ranking of enrichment in promoter acetylation and in gene expression (Table S2). We found that genes in H3K9ac and H3K27ac lists were strongly associated with proteins that function in transcriptional regulation (Table S3) and included genes that we previously identified as regeneration inhibitory (H3K9ac: *Cited2*, *Cbx4*, *Ngfr*; H3K27ac: *Cited2*, *Cbx4*, *Adrb2*, *Bcor*). We therefore investigated histone acetylation changes for the 29 genes that were classified as regeneration permissive or inhibitory using Nanostring gene expression data (Voss et al., 2021), which showed that romidepsin increased the expression of inhibitory genes and decreased the expression of permissive genes. Most of the permissive (ten out of 13) and inhibitory (15 out of 16) genes showed higher H3K9ac and H3K27ac in promoters for R-6HPA in comparison to C-6HPA; however, acetylation changes were higher for genes that were upregulated by romidepsin, consistent with the analyses above (Fig. 7A,B; Fig. S3A,B). Also, the bivariate ranks for regeneration-inhibitory genes were all higher than the ranks for permissive genes (Fig. 7C). These results implicate romidepsin-mediated promoter acetylation in the expression of regeneration-inhibitory genes.

### Proteome-level effects of romidepsin on lysine acetylation

HDACs remove acetyl from lysine residues within histone and non-histone proteins. To assess protein acetylation more comprehensively, we performed nanoscale liquid chromatography-tandem mass spectrometry (Nano LC-MS/MS) to quantify the abundances of acetylated versus non-acetylated peptides from total protein samples collected at 0-HPA, C-6HPA, and R-6HPA. A total of 4589 modified proteins and 18,961 quantitative acetylation sites were detected among samples (Table S4). Statistical (*t*-test probability<0.05) and fold-change (FC>1.5) criteria were used to identify residues with increased or decreased acetylation. We first examined differentially acetylated sites that were identified in both the 0-HPA versus C-6HPA and 0-HPA versus R-6HPA contrasts; presumably these sites are not regulated by class I HDAC activity. A total of 343 sites that were identified as significantly increased or decreased in the 0-HPA versus C-6HPA contrast were also found to be significant in the 0-HPA versus R-6HPA contrast (Table S4). None of these sites were inversely regulated between the contrasts. Upregulated and downregulated sites mapped to 94 and 131 unique proteins, respectively. Sixty-six upregulated sites were identified for seven different hemoglobin proteins (Hba, Hba1, Hbb, Hbe, Hbg1, Hbg2, Hbz) and three fibrinogens (Fga, Fgb, Fgg), with 11-12 acetyl-lysine sites identified within each fibrinogen paralog (Table S4). These and other proteins significantly enriched Gene Ontology biological processes (BP-GOs) associated with erythrocyte development, blood coagulation, fibrinolysis, and wound healing. Other BP-GOs that were enriched for upregulated and downregulated sites included chromatin organization, regulation of gene expression, and regulation of metabolic process (Table S5). The identification of the same differentially acetylated sites for C-6HPA and R-6HPA samples validated the Nano LC-MS/MS approach across biological samples and showed that many sites within wound-healing proteins are regulated by non-class I HDACs.

**Fig. 8.**
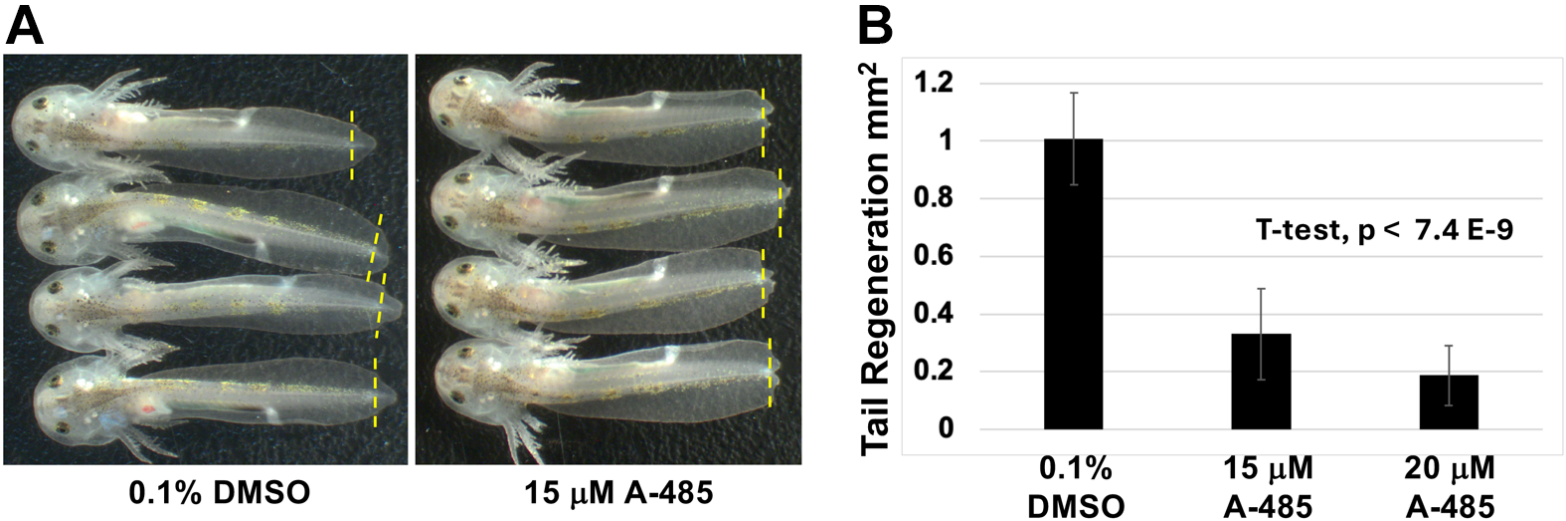
**Effect of A-485 on axolotl embryo tail regeneration.** (A) Representative images of tail regeneration at 7 days post amputation (DPA) for 0.1% DMSO- and 15 μM A-485-treated embryos. The dashed yellow lines indicate the plane of amputation. (B) The amount of regenerated tail tissue at 7 DPA for embryos that were treated with 0.1% DMSO (*N*=11), 15 μM A-485 (*N*=10), and 20 μM A-485.

In the contrast to 6HPA samples, more than twice as many upregulated sites (291 versus 132) were identified for R-6HPA (Table S4). The upregulated and downregulated sites mapped to 245 and 105 unique proteins, respectively. These proteins enriched a diversity of BP-GOs, including metabolic process, RNA metabolic process, translation, wound healing, regulation of gene expression, and chromatin remodeling (Table S5). The list of over 100 genes enriching the regulation of gene expression BP-GOs included *Hoxd10*, *Stat3*, *Cbx8*, *Runx1*, *Arid3a*, *Aff4*, *Spen*, *Kmt2b*, *H2ax*, *H3.1*, *Jade3*, *Purb*, *Smarcc1*, *Smarca5*, *Crebbp*, and *Ep300.* Given the possibility that romidepsin affects the expression of regeneration-inhibitory and -permissive genes via differential acetylation of one or more transcription factors, we prioritized Crebbp/Ep300 for further study. Crebbp/Ep300 are highly conserved paralogs that have multiple protein domains to enable transcription factor binding and lysine acetylation (Chan and La Thangue, 2001). We observed increased acetylation for a site in Crebbp (XP_069502924_1_K73) in response to romidepsin, while a site in Ep300 (XP_069510525_1_K2055) showed a decrease. Previously, we hypothesized that romidepsin may alter injury transcriptional responses to hypoxia that are mediated by Ep300/Crebbp (Baddar et al., 2021). To further explore this hypothesis, we treated tail-amputated embryos with A-485, a small molecule that blocks Crebbp/Ep300 acetyltransferase activity (Lasko et al., 2017). We found that 15 and 20 μM A-485 potently inhibited regeneration (Fig. 8). To determine the effect of A-485 on gene expression, RNA sequencing (RNA-SEQ) was performed to identify differentially expressed genes between 0.1% DMSO- and 20 μM A-485-treated embryos at 24HPA. A total of 6189 differentially expressed transcripts were identified, with the majority (*N*=3588) showing significantly higher expression in the control/DMSO sample (Table S6). GO analyses of upregulated and downregulated genes identified 314 and 2592 significantly enriched BP-GOs, respectively (Table S7). In comparing the two lists of BP-GOs, we noticed that A-485 decreased genes that uniquely enriched ontologies associated with cell signaling and regeneration. For example, downregulated genes enriched multiple cell-signaling pathways (PDGFR, WNT, RA, BMP, SMAD, MAPK, VGF, Il11, GDNF, NGF, CNTF, IGF, FGF, and VEGF) and regeneration BP-GOs (regeneration, tissue regeneration, animal organ regeneration, neuron projection regeneration, liver regeneration, skeletal muscle tissue regeneration, axon regeneration, and bone regeneration); no significantly enriched cell signaling or regeneration BP-GOs were identified for the upregulated gene list. Instead, 32 BP-GOs associated with cell cycle process, cell cycle regulation, and cell cycle transition were significantly and uniquely enriched for the upregulated gene list. Also, for this list, no BP-GOs associated with cell cycle arrest, growth arrest, or apoptosis were enriched. These results strongly implicate Crebbp/Ep300 in the regulation of biological processes that are required for tail regeneration.

We next compared the effects of A-485 and romidepsin on the expression of regeneration-permissive and -inhibitory genes from Voss et al. (2021). These genes were previously classified as permissive versus inhibitory based on expression values measured at 6HPA and 12HPA. At 24HPA, which is when we evaluated the effect of A-485 treatment on transcription, we were able to identify fold change values between romidepsin- and DMSO-treated embryos for 28 of the 29 originally classified genes. All 12 of the previously classified regeneration-permissive genes, and 13 of 16 of the previously classified regeneration-inhibitory genes, showed expression differences consistent with romidepsin treatment effects at 6HPA and 12HPA. We next compared the fold change values of these genes to those calculated between A-485- and DMSO-treated embryos. Of the 15 genes that were downregulated by romidepsin at 24HPA, ten were also downregulated by A-485 (Fig. S4). We note that *Has2*, which is required for tail regeneration (Voss et al., 2021), was downregulated by 1.26- and 3.95-fold by romidepsin and A-485, respectively. Other genes that are normally upregulated during regeneration (e.g. *Lep*, *Krt17*, *Cyp26b1*) were similarly downregulated by romidepsin and A-485. In contrast, A-485 did not increase regeneration-inhibitory genes identified by romidepsin treatment; only one of 13 genes was slightly increased by A-485; *Nr4a1* was increased 2.0-fold by romidepsin, but only 0.69-fold by A-485. Thus, while romidepsin and A-485 decreased transcription of key regeneration-permissive genes, these two chemicals altered different sets of regeneration-inhibitory genes. These results suggest a requirement for HDAC class I and Ep300/Crebbp activities in the regulation of regeneration-permissive genes.

## DISCUSSION

Studies using a variety of animal models and HDACi have shown that HDAC activity is required for successful appendage regeneration (Tseng et al., 2011; Taylor and Beck, 2012; Voss et al., 2019, 2021; Wang et al., 2019; Arbach et al., 2022). HDACi are thought to inhibit regeneration through changes in protein lysine acetylation dynamics that impinge on transcriptional regulation. While aberrant patterns of transcription have been documented for appendage regeneration in response to HDACi, the underlying effects on lysine acetylation have not been described. Studies of yeast and cell lines have shown that HDACi shifts acetylation dynamics in the direction of acetyltransferase activity, which increases lysine acetylation of histones (Kurdistani and Grunstein, 2003; Wang et al., 2009b). Increased histone acetylation relaxes chromatin and makes gene promoters more accessible to transcriptional regulation. HDACi also increases acetylation and alters the functions of non-histone proteins, including transcription factors that activate and repress transcription (Smith, 2008). Because HDACi may complexly impact transcriptional regulation through changes in histone and non-histone acetylation, both mechanisms were investigated in this study.

In the axolotl, romidepsin inhibits regeneration and the transcription of regeneration-permissive genes that are normally expressed during tail regeneration, while simultaneously upregulating the expression of genes that are predicted to inhibit regeneration (Voss et al., 2019, 2021). We extended this body of work by using genomic and proteomic approaches to detail romidepsin-mediated lysine acetylation changes in histone and non-histone proteins at the outset of tail regeneration. Below, we discuss five primary results of our study. (1) Romidepsin increased lysine acetylation genome wide, countering a deacetylation response that may typify appendage regeneration. (2) Histone acetylation and gene expression were only weakly positively associated when considering all genes with evidence of acetylated promoters. (3) Genes that showed high promoter acetylation and gene expression in response to romidepsin included previously identified regeneration-inhibitory genes and newly discovered transcription factors. (4) Romidepsin altered lysine acetylation of non-histone proteins, including Ep300/Crebbp, which we show are required for tail regeneration and expression of regeneration-associated genes. (5) Hemostatic proteins showed romidepsin-independent changes in lysine acetylation that are predicted to be adaptive for wound healing. We conclude by addressing caveats and limitations of our study.

### Romidepsin increased histone lysine acetylation genome wide

In our initial screen of histone types and subtypes, we observed acetylation changes across multiple histone PTMs for control samples that indicate typical or normal responses to amputation injury. These changes were observed for highly and lowly abundant histone PTMs that are known from other studies to regulate chromatin structure and function. H3K9ac and H3K27ac, PTMs that are associated with active transcription, showed a decrease in acetylation at 6HPA, a pattern that was detected by mass spectrometry, ChIP-SEQ, and CUT&RUN. Romidepsin countered the control/normal deacetylation response observed in promoter, gene body, and intergenic regions of the genome by globally increasing histone acetylation. HDACi increase acetylation because they disrupt the dynamics of acetylation change mediated by histone acetyltransferases (HATs) and HDACs, as these enzymes work in opposition to remove and add acetyl-lysine to establish chromatin contexts that effect gene expression. HDACi shift the HAT/HDAC equilibrium towards increased lysine acetylation, globally increasing chromatin accessibility and thus profoundly altering chromatin structure, in opposition to what normally occurs after amputation injury. A genome-wide decrease in acetylation is consistent with a requirement for HDAC in regeneration, a requirement that has been established in multiple regeneration-competent species (Tseng et al., 2011; Taylor and Beck, 2012; Li et al., 2017; Voss et al., 2019, 2021; Wang et al., 2019; Zondag et al., 2019; Arbach et al., 2022).

Our study establishes an important new tool for epigenetic studies of the axolotl. In contrast to studies that used pan-HDACi to broadly inhibit multiple HDAC enzymes (e.g. TSA, SAHA, vorinostat), romidepsin is a specific HDAC class I inhibitor. Thus, our findings implicate HDAC class I enzymes (HDAC1, HDAC2, and HDAC3) as regulating genome-wide patterns of H3K9ac and H3K27ac in the axolotl genome. Chemicals other than romidepsin are available to differentially target HDAC enzymes to elucidate their unique and combinatorial effects on lysine acetylation. Our results establish romidepsin as a reproducible and robust tool for inducing histone acetylation in future studies of axolotl tail regeneration, and perhaps other axolotl models of regeneration.

### Histone acetylation and gene expression are only weakly positively associated

Histone acetylation dynamics are associated with changes in chromatin accessibility and transcription. An HDACi-mediated, genome-wide increase in histone acetylation would be expected to increase chromatin accessibility at thousands of gene promoters and alter transcriptional activity. To test for an association between histone acetylation and transcription, we first analyzed a large number of genes with evidence of acetylation, and then analyzed a smaller number of genes that were identified as regeneration permissive or inhibitory from previous studies. When analyzing thousands of genes with acetylated promoters, we detected a weak positive association for genes expressed in control samples, and a slightly higher positive association for genes expressed in romidepsin-treated samples. Similarly, we observed a weak positive association for the smaller set of regeneration-permissive and -inhibitory genes. We reason that acetylation and gene expression are only weakly associated because promoter acetylation contributes to the transcriptional regulation of some but not all genes, and because transcription is modified by other pathways acting in parallel. The magnitude of increase in promoter acetylation under romidepsin was observed to be significantly higher for upregulated genes, including previously identified regeneration-inhibitory genes and newly discovered genes that were enriched for transcription regulatory functions. These genes are expressed when romidepsin exceeds a threshold concentration (Voss et al., 2021), supporting the idea that histone acetylation and transcription are dose-dependently regulated. HDACi-mediated upregulation of genes like *Cited2*, which showed increased promoter acetylation, may affect the early response to hypoxia and engender a reactive oxygen environment that is inhibitory to regeneration (Baddar et al., 2021). Mechanisms that regulate the sensitivity of oxygen sensing may be key to appendage regeneration (Mui et al., 2026; Tsissios et al., 2026).

A mechanistic explanation for the regulation of regeneration-permissive genes is less clear as these genes generally showed increased acetylation with either increased and decreased gene expression. It is possible that promoter acetylation equilibrium (determined by the competitive activities of acetyltransferases and deacetylases) or transient deacetylation, is required for the expression of regeneration-permissive genes; this is consistent with the global deacetylation response observed for control samples at 6HPA and the expression of permissive genes at low, sub-threshold romidepsin concentration. Alternatively, these genes may require HDAC activity within chromatin complexes that mediate promoter-enhancer interactions or transcriptional elongation of promoter pause-released genes (Greer et al., 2015; Quaas et al., 2022). Additional studies are needed to fully understand how HDAC activity regulates alternative transcriptional and regeneration responses.

### Romidepsin-mediated changes in non-histone protein acetylation

Transcriptional regulation may depend upon non-histone proteins, including transcription factors, that are differentially activated or repressed by lysine acetylation (Smith, 2008). Romidepsin not only increased acetylation of histones but generally increased protein acetylation overall. Mass spectrometry analysis of acetylated peptides revealed approximately twice as many acetylated lysine peptides for romidepsin-treated versus control samples at 6HPA, including transcription factors that may regulate regeneration-associated genes. For example, lysine residues within Crebbp and Ep300 were differentially acetylated in the presence of romidepsin, a finding that led us to investigate the requirement of these proteins for regeneration. Crebbp/Ep300 regulate transcription by acetylating histone and non-histone proteins. The protein targets of Crebbp/Ep300 are predominantly nuclear proteins that function in transcription and chromatin regulation; however, other targets modulate signaling cascades that are integral for development, cell cycle regulation, and regeneration (Di Giovanni et al., 2006; Tedeschi et al., 2009; Spange et al., 2009; Weinert et al., 2018). For example, Crebbp/Ep300-mediated acetylation stabilizes the Tp53 protein for transcriptional activation of genes associated with cell cycle arrest and apoptosis (Ito et al., 2001). However, in a very different biological context, Crebbp/Ep300 acetylation of Tp53 activates genes associated with axon regeneration and outgrowth (Di Giovanni et al., 2006; Tedeschi et al., 2009). Using A-485 to block the acetyltransferase activity of Crebbp/Ep300, we inhibited tail regeneration and dramatically altered transcription of candidate target genes. A-485 inhibited many of the same regeneration-permissive genes that were previously identified using romidepsin, but not the same set of regeneration-inhibitory genes. This is interesting because romidepsin and A-485 are expected to inversely affect protein acetylation. In contrast to romidepsin-induced protein hyperacetylation, which our results clearly document, A-485 is predicted to reduce acetylation of Crebbp/Ep300 targets, including histone and non-histone proteins. It is possible that regeneration-permissive gene expression depends upon both Crebbp/Ep300 and HDAC activities, while regeneration-inhibitory gene expression is associated with histone hyperacetylation, a state that is induced by romidepsin but perhaps not by A-485. This hypothesis is consistent with our observation of higher promoter acetylation for regeneration-inhibitory versus -permissive genes. The unique upregulation of cell cycle progression and mitosis genes by A-485 is consistent with a decrease in Tp53 stability; however, we did not identify Tp53 as a differentially acetylated protein. Further studies are needed to determine the independent and combined effects of HDAC and Crebbp/Ep300 on transcription during tail regeneration.

### Romidepsin-independent changes in non-histone protein acetylation

Although our study focused on the effects of romidepsin on protein acetylation, romidepsin-independent effects were also observed. Acetylation was increased in control and romidepsin-treated samples for wound healing proteins, including multiple fibrinogens and hemoglobin. These presumably class I HDAC-independent acetylation changes may be important for hemostasis. Crebbp/Ep300-mediated protein acetylation is essential for platelet functions, including activation and aggregation (Aslan et al., 2015). Additionally, fibrinogen acetylation increases fiber density, a structural change that makes fibrin clots more porous and susceptible to fibrinolysis (Caspary, 1956; Nencini et al., 2024). The formation of a transient fibrin clot may be a hallmark of regeneration-competent species as it would seemingly enable a more rapid transition from hemostasis to early regeneration responses. The acetylation of hemoglobin is predicted to alter oxygen binding affinity, which may be adaptive in hypoxic, injury environments (Manning and Manning, 2001). These hypotheses await further study.

### Caveats

In closing, we note several caveats of our study. First, we examined relatively few PTMs and post-injury time points for protein acetylation changes. More comprehensive sampling of PTMs would better capture the dynamics of protein PTM during regeneration; of course, this is not trivial as it would require optimization of reagents and protocols for each PTM. Although we reported previously that romidepsin altered similar changes in transcription across cell types (Voss et al., 2021), in this study we analyzed bulk tissue samples and therefore did not detail cell-specific changes in acetylation or transcription. Ongoing efforts to develop single-cell epigenetic approaches should eventually allow measurement of site-specific changes in histone modification in individual cell types within complex tissues. Methods used to perform epigenetic analyses of large genomes are still very much in their infancy and can likely be improved. Similarly, there have been very few proteomic studies of amphibians. Although we singled out Crebbp/Ep300 for further study, we did not directly test the function of differentially acetylated lysines within these proteins for an effect on regeneration or transcription. In the future, it will be important to genetically replace codons specifying lysine residues for alternative amino acids to directly test the effect of lysine acetylation on Crebbp/Ep300 protein function. For example, the differentially acetylated lysine sites that were identified for *Runx1* in this study have been replaced with arginine residues in Runx1 recombinant proteins to show a requirement for transcription factor activity (Yamaguchi et al., 2004). Even with these caveats, our study provides new data and novel insights about histone and non-histone protein acetylation during tissue regeneration.

## MATERIALS AND METHODS

### Embryo husbandry and chemical treatment

The use of pre-feeding-stage axolotls does not require a protocol approved by the Institutional Animal Care and Use Committee (IACUC) at University of Kentucky; however, embryos used in this study were treated according to the same ethical standards that apply to feeding axolotls that are covered by IACUC protocol 2020-3449. Axolotl embryos (RRIDs: AGSC_100E, AGSC_101E, AGSC_102E, AGSC_103E) were obtained from the Ambystoma Genetic Stock Center (AGSC RRID: SCR_006372). Embryos were housed initially in 2-l glass bowls containing reverse osmosis water with 1.38 g NaCl, 80 mg MgSO4, 40 mg CaCl_2_, and 20 mg KCl per liter) in a room maintained at 17-18°C and illuminated with cool white fluorescent lighting (12 h light/12 h dark).

### Tissue collection for histone mass spectrometry

Developmental stage 42 (Bordzilovskaya et al., 1989) embryos were anesthetized in 0.02% benzocaine and then administered tail amputations to remove 2 mm of the distal tail tip. After amputation, 300 embryos were randomly assigned to five groups (60 per group): 0-HPA, C-6HPA, R-6HPA, C-24HPA, R-24-HPA. 0-HPA embryos immediately received a second amputation that removed 1 mm of the distal tail stump. These 1 mm tail pieces were combined and immediately flash frozen on dry ice. Embryos in the other two groups were reared independently in 12-well microtiter plates in 2 ml of either 0.1% DMSO (C-6HPA) or 10 μM romidepsin (R-6HPA). After 6 or 24 h of treatment, 1 mm of the distal tail tip was collected from each embryo and pooled to form samples (one per group).

### Mass spectrometry quantification of histone acetylation PTMs

Distal tail tissue was collected from axolotl embryos (*N*=375) according to the method described above. Five tissue samples (0-HPA, C-6HPA, R-6HPA, C-24HPA, and R-24HPA) were prepared by pooling 75 tail tips per sample. Histone extraction and mass spectrophotometry were performed by a commercial vendor (Active Motif). Histones were acid extracted, derivatized via propionylation and digested with trypsin, and newly formed N-termini were propionylated as previously described (Garcia et al., 2007). Each sample was resuspended in 50 μl of 0.1% TFA/mH_2_O, and 3 μl was injected with three technical replicates in a Thermo Fisher Scientific TSQ Quantum Ultra mass spectrometer coupled with an UltiMate 3000 Dionex nano-liquid chromatography system. The data were quantified using Skyline (MacLean et al., 2010) to obtain the percent of each histone modification within the total pool of each corresponding amino acid residue. Z-scores were calculated by subtracting the average percent abundance of each PTM from the mean across samples and then dividing this value by the standard deviation of average percent abundance across samples.

### ChIP-SEQ

Distal tail tissue was collected from axolotl embryos (*N*=2430) according to the method described above. Six tissue samples (0-HPA, C-6HPA, and R-6HPA) were prepared by pooling 540 tail tips per H3K9ac chromatin preparation and 270 tail tips per H3K27ac chromatin preparation. An additional 540 tail tips were collected for an input control chromatin preparation. ChIP-SEQ libraries were prepared by a commercial vendor (Zymo) using a proprietary library preparation protocol. The validated antibodies that were used included anti-H3K27ac polyclonal antibody (Abcam ab4729, Lot no. GR3231937-1; RRID:AB_2118291), anti-H3K9ac antibody (Millipore 07-352, Lot no. 3314305; RRID:AB_310544), and negative controls anti-IgG rabbit antibody (Diagenode C15410206, Lot no. RIG001AK; RRID:AB_2722554) and anti-IgG mouse antibody (Millipore 12-371, Lot no. 2951852; RRID:AB_145840). The ChIP-SEQ assays were performed using 5 μg of chromatin and 2 μg of antibody. ChIP-SEQ libraries were quantified by Qubit and quantitative PCR, and diluted to 2 nM before being multiplexed and run on a Next-Gen Sequencing platform (Illumina HiSeq). The ChIP- SEQ data were submitted to NCBI Sequence Read Archive (SRA; PRJNA1399472). Sequenced data were processed using the Zymo bioinformatics pipeline adapted from nf-core ChIPseq pipeline (https://github.com/Zymo-Research/service-pipeline-documentation/blob/master/docs/how_to_use_ChIPseq_report.md). For each sample, the following pipeline steps were performed. Single-end reads were trimmed with Trim Galore v0.5.0, aligned to the genome reference (GCA_002915635.3: AmbMex60DD) and filtered to remove duplicates, discordantly mapped reads, reads that mapped to multiple genomic positions, and alignments with >4 mismatches (Samtools v1.9, Picard v2.19.0, BamTools v2.5.1). Narrow peaks for each sample were called using MACS2 v2.1.2 based on all uniquely mapped and filtered reads, and evaluated using featureCounts v1.6.4 (Liao et al., 2014) by calculating the fraction of all mapped reads that fall into the called peak regions. A read must overlap a peak by at least 20% to be counted. Genome-wide read coverage was computed and reads per million (RPM) normalized by scaling to 1 million mapped reads with BEDTools v2.27.1 and bedGraphToBigWig.

To compare acetylation between samples, we combined peaks from all three samples to form a union set of peak intervals for H3K9ac and separately for H3K27ac, such that two individual peaks were merged if they overlapped by at least half of the combined peak length.

Promoter regions, gene bodies and intergenic regions were identified based on the annotation AmexT_v47 for AmbMex60DD reference (https://www.axolotl-omics.org/assemblies) filtered to maintain primary isoforms. To assess acetylation signals in a genomic region, which can be influenced by the number of cells with acetylated histone marks in the region and the affinity of antibody binding, we used the sum of normalized coverage values (mass) over promoter or peak intervals to perform comparisons between samples. Acetylation mass values for gene bodies and intergenic regions were computed as the total mass of the peak union within the regions, while for promoters, the entire length of the interval 2 kb upstream and 2 kb downstream of the TSS was used. Acetylation profiles near TSSs were generated using deepTools (version 3.5.6) with missingDataAsZero parameter for computeMatrix reference-point tool and visualized using plotProfile.

### CUT&RUN

Distal tail tissue was collected from axolotl embryos (*N*=1200) according to the method described above. Tissue samples (0-HPA, C-6HPA, and R-6HPA) were prepared by pooling 240 tail tips per H3K27ac chromatin preparation. Additional pools of 240 tail tip tissue pieces were collected for positive and negative control reactions per instructions in the CUTANA Cut and Run Kit (Epicypher Cat. no. 14-1048). Nuclei were isolated using freshly prepared nuclei isolation buffer (NIB) (15 mM Tris-HCl, 2 mM EDTA, 80 mM KCl, 20 mM NaCl, 0.04% v/v 2-ME, 0.5 mM Spermine, 0.2% w/v bovine serum albumin, 0.05% v/v Triton X-100). Tail tips and 250 μl of NIB were added to a 2 ml dounce homogenizer on ice. The tissue was homogenized using both tight and loose pestles, and then the dounce was rinsed with 250 μl more NIB. The total 500 μl of homogenate was passed through 100-, 70-, then 40-μm cell strainers (Corning). Nuclei quality and quantity were assessed on a disposable hemocytometer using Trypan Blue staining. Approximately 100,000 nuclei per treatment were incubated with Concanavalin A (ConA) beads and assessed for bead binding. Nuclei were then incubated overnight at 4°C with ConA beads and 0.5 µg of antibody H3K27ac (Epicypher Cat. no. 13-0059; RRID:AB_3712075), 1 μl of H3K4me3 antibody (Epicypher Cat. No 13-0060; no RRID registered) for the positive control and 1 μl of non-immune IgG for the negative control. The following day, antibody-labeled chromatin was bound by pAG-MNase and then released by calcium activation of MNase. A reaction stop buffer containing 0.1 ng of *Escherichia coli* spike-in DNA was then added to halt MNase activity. CUT&RUN-enriched DNA was purified by SPRIselect bead purification (Beckman Coulter). Samples were quantified using a Qubit fluorometer and dsDNA HS assay kit (Thermo Fisher Scientific).

CUT&RUN libraries were prepared using a CUTANA™ library prep kit (Epicypher Cat. No. 14-1001). The entire volume of each DNA sample was used for library preparation. Prepared libraries were quantified and analyzed for fragment distribution by staff in the Oncogenomics Shared Resource of the University of Kentucky Markey Cancer Center, using the D1000 ScreenTape assay for TapeStation (Agilent). Each library was diluted to 4 nM, denatured with NaOH, and diluted to a loading concentration of 12 pM. The prepared CUT&RUN libraries were first sequenced in house on an Illumina MiSeq, producing 18, 16 and 12 million paired reads for C-0HPA, C-6HPA and R-6HPA. Additional sequencing was performed by Admera Health LLC, delivering 53, 80, and 54 million reads per library, respectively. All sequence data were submitted to NCBI SRA (PRJNA1399509). An analysis pipeline nf-core/cutandrun v3.2.2 (Meers et al. 2019) implemented in Nextflow v25.04.6 was used for the initial processing of sequencing reads, including adapter trimming, alignment to the reference genome using Bowtie2 v2.4.4, quality filtering (mapping quality >20), and duplicate marking. Read coverage was calculated using bedtools genomecov v2.27.1 with the –fs option forcing use of mean fragment size. Coverage depth for each sample was RPM normalized using a scaling factor of 10^6^ divided by the number of reads in a filtered alignment file. Overall H3K27ac acetylation was calculated using the union of peak intervals identified from the H3K27ac ChIP-SEQ analysis. Acetylation mass values for promoters, gene bodies and intergenic regions, as well as acetylation profiles near TSSs, were calculated in the same way as for the ChIP-SEQ data.

### Relationship between histone acetylation and gene expression

snRNA-SEQ data (Voss et al., 2021) for each sample were analyzed using Cell Ranger (v.7.1.0), reference genome GCA_002915635.3 (AmbMex60DD) and the same annotation that was applied to define genomic regions. Aggregated results from Cell Ranger were used to perform pairwise differential analyses between samples in Loupe Browser 9.0.0. To compare promoter acetylation and transcription changes, we filtered out genes with overlapping promoters and genes with promoter acetylation mass <200 in all samples. For genes differentially expressed between C-6HPA and R-6HPA with *P*-value<0.1, we assigned differential expression ranks as (log_2_FC−min)/(max−min), where ‘min’ and ‘max’ represent the minimum and maximum log_2_FC values among these genes; thus, all ranks fall within the [0,1] interval. After defining the differential acetylation rank in a similar manner, we computed for each gene a bivariate rank as the mean of the differential expression rank and the differential acetylation rank. Two-tailed *t*-tests between groups of upregulated and downregulated genes, and between regeneration-permissive and regeneration-inhibitory genes, were performed in JMP (v.19.0.1 Student Edition).

### Nano LC-MS/MS quantification of acetylated lysine peptides

A total of 162 larval axolotls (3 cM total body length) were used for tissue collection. Individuals were anesthetized in 0.02% benzocaine, and tail amputations were performed to remove 2 mm of the distal tail tip. After amputation, individuals were randomly assigned to three groups (54 per group): 0-HPA, C-6HPA, and R-6HPA. 0-HPA individuals immediately received a second amputation that removed 1 mm of the distal tail stump. These 1 mm tail pieces were immediately flash frozen on dry ice and combined to form samples (nine pieces per replicate, six replicates in total). Individuals in the other two groups were reared independently in plastic trays containing 3 ml of either 0.1% DMSO (C-6HPA) or 10 μM romidepsin (R-6HPA). After 6 h, 1 mm of the distal tail tip was collected from each individual and pooled to form six biological replicates for each treatment. Overall, 18 tissue samples were prepared. Protein extraction, protein digestion, acetylated peptide enrichment, and Nano LC-MS/MS analysis were performed by a commercial vendor (Creative Proteomics). Because the protein level was low for one of the 0-HPA replicates, it was combined with another sample to yield a single sample. Peptide samples were separated by chromatography, and mass spectrometry data were collected using the ddaPASEF mode of the timsTOF Pro2 mass spectrometer (Bruker). FragPipe v21.1 (Yu et al., 2020) was used for protein and PTM identification and quantitative analysis of mass spectrometry data. To assign peptides to proteins, peptides were searched against axolotl NCBI RefSeq proteins using Blastp. Statistical (*t*-test probability<0.05) and fold-change (FC>1.5) criteria were used to identify upregulated and downregulated acetylation sites.

### A-485 treatment of axolotl embryos and RNA-SEQ analysis

Developmental stage 42 (Bordzilovskaya et al., 1989) embryos were anesthetized in 0.02% benzocaine and then administered tail amputations to remove 2 mm of the distal tail tip.

Embryos were treated for 7 days in either 0.1% DMSO (*N*=11), 15 μM A-485 (*N*=10), or 20 μM A-485 (*N*=12) and then imaged using an Olympus dissecting microscope and DP28 camera. Additional embryos were subsequently treated after amputation in either 0.1% DMSO or 20 μM A-485 for 24 h. At 24HPA, 1 mm tail pieces were collected from embryos and pooled to prepare three replicate samples per treatment (12 tail tips per replicate). These samples were immediately flash frozen on dry ice and stored in a −80°C freezer until the time of RNA isolation. RNA was isolated using TRIzol (Invitrogen) and syringe needles for initial tissue disruption, and then following instructions included with a Monarch Total RNA Isolation Kit (NEB). RNA samples were submitted to a commercial vendor (Novogene) to generate approximately 25-30 million raw reads per sample on an Illumina sequencer. RNA-SEQ reads were aligned to the reference genome using HISAT2 v2.2.1 (Kim et al., 2019). Gene expression estimates were obtained from these alignments using StringTie v2.2.3 (Pertea et al., 2015), and differential expression analysis was conducted using the DEseq2 R package v1.42.1 (Love et al., 2014). The RNA-SEQ data were submitted to NCBI Gene Expression Omnibus (GEO; GSE315749).

### Gene Ontology analyses

Gene Ontology analyses were performed using the Panther statistical over-representation test (Mi et al., 2019). The complete set of BP-GOs was used, and testing was performed using the Fisher’s exact test with false discovery rate correction.

## Supplementary Material



10.1242/biolopen.062726_sup1 Supplementary information

Table S1. Summary statistics for ChIP-seq data.

Table S2. Changes in gene expression and promoter acetylation for all expressed genes with evidence of acetylation (promoter mass > 200 in at least one sample). Bivariate rank data for genes differentially expressed between C-6HPA and R-6HPA (p-value < 0.1).

Table S3. Panther protein classifications that were significantly enriched by genes with the highest (top 5%) bivariate ranking scores for promoter acetylation and gene expression.

Table S4. Differentially acetylated proteins identified between contrasts of 0-HPA, C-6HPA, and R-6HPA samples.

Table S5. Biological process ontologies that were significantly enriched by differentially acetylated proteins identified between contrasts of 0-HPA, C-6HPA, and R-6HPA samples.

Table S6. Differentially expressed genes identified between DMSO and A-485 treated embryos at 24 HPA.

Table S7. Biological process ontologies that were significantly enriched by the list of genes that were significantly differentially expressed between DMSO and A-485 treated embryos at 24 HPA.
