# Supplementary material for: Histone and non-histone protein acetylation in a tail regeneration model (Ambystoma mexicanum)
Source: Biol Open. 2026 Jul 14;15(7):bio062726. doi: 10.1242/bio.062726 (PMC13446560; doi:10.1242/bio.062726)
Supplement: Supplementary file 1 [file biolopen-15-062726-s1.pdf]

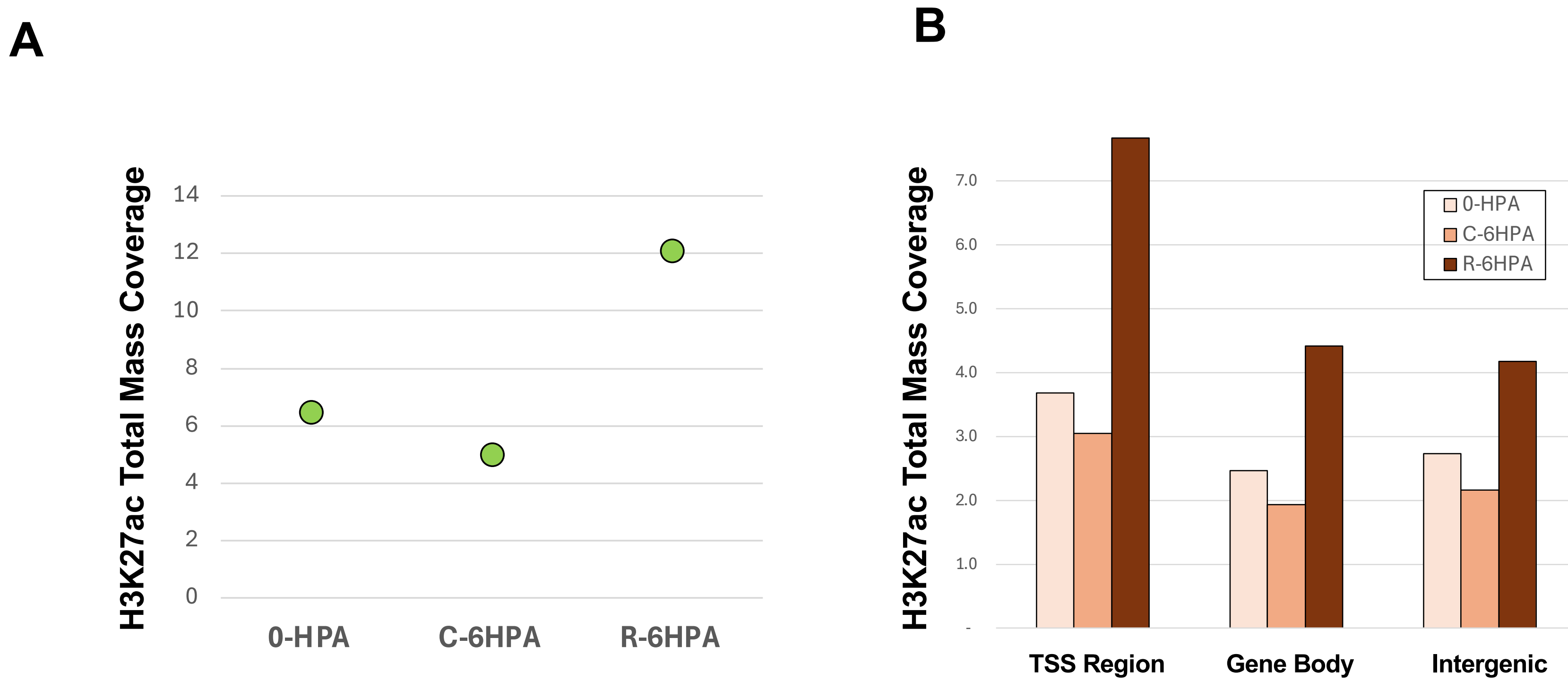

Fig. S1. Genomic patterns of H3K27ac coverage at the outset of axolotl embryo tail regeneration. A) Total mass of CUT&RUN coverage across the genome for 0-HPA, C- 6HPA, and R-6HPA samples. Total mass was calculated for intervals identified by ChIP-SEQ. B) Total mass of CUT&RUN coverage for different genomic features, including promoters (+/- 2Kb of TSS), gene bodies, and intergenic regions, calculated for the same intervals as in ChIP-SEQ analysis.

Biology Open • Supplementary information

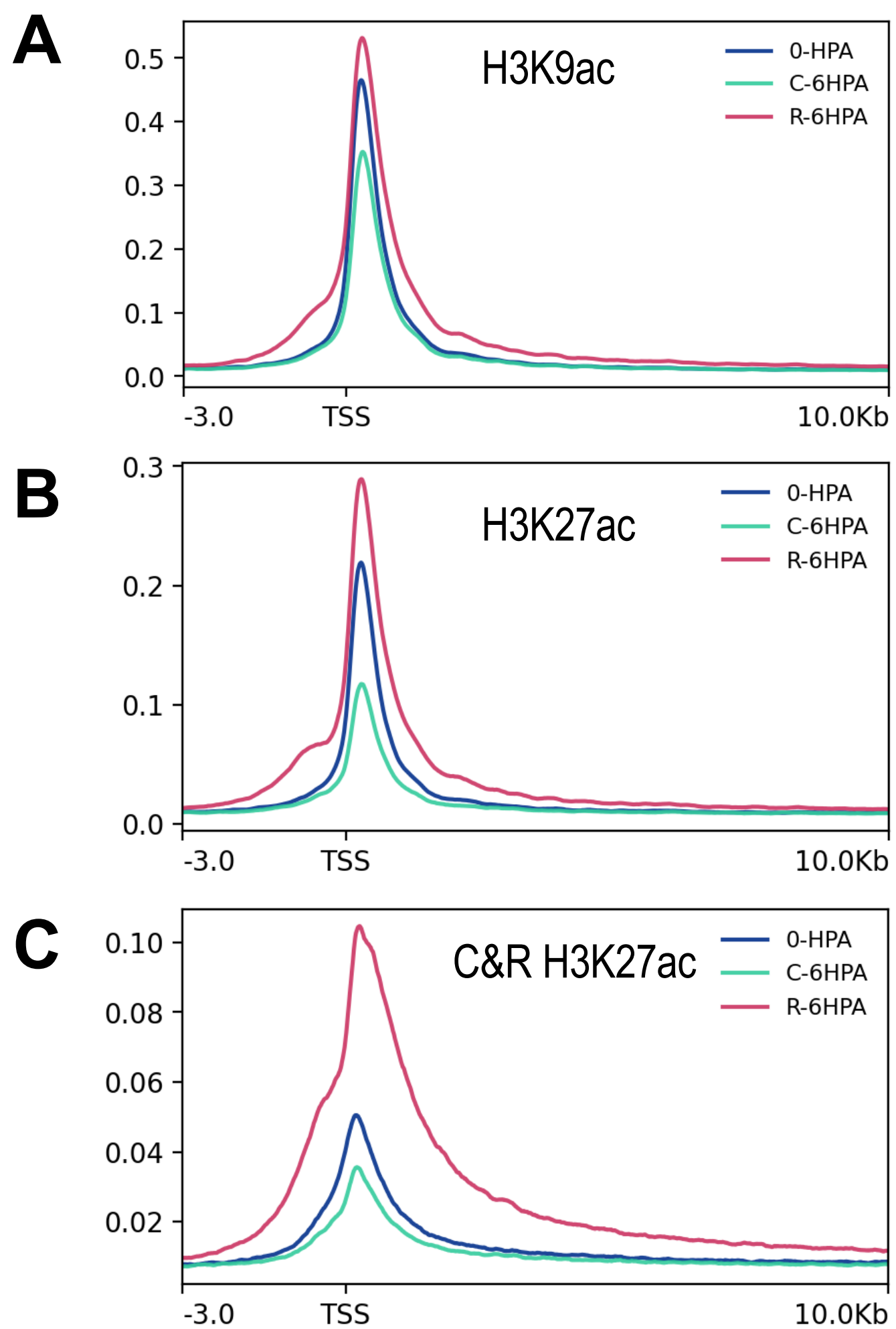

Fig. S2. Romidepsin-mediated spreading of acetylation in promoters. Plots show mean, normalized coverage of A) ChIP-SEQ H3K9ac, B) ChIP-SEQ H3K27ac, and C) CUT&RUN H3K27ac upstream (-3 Kb) and downstream (+10 Kb) of TSS.

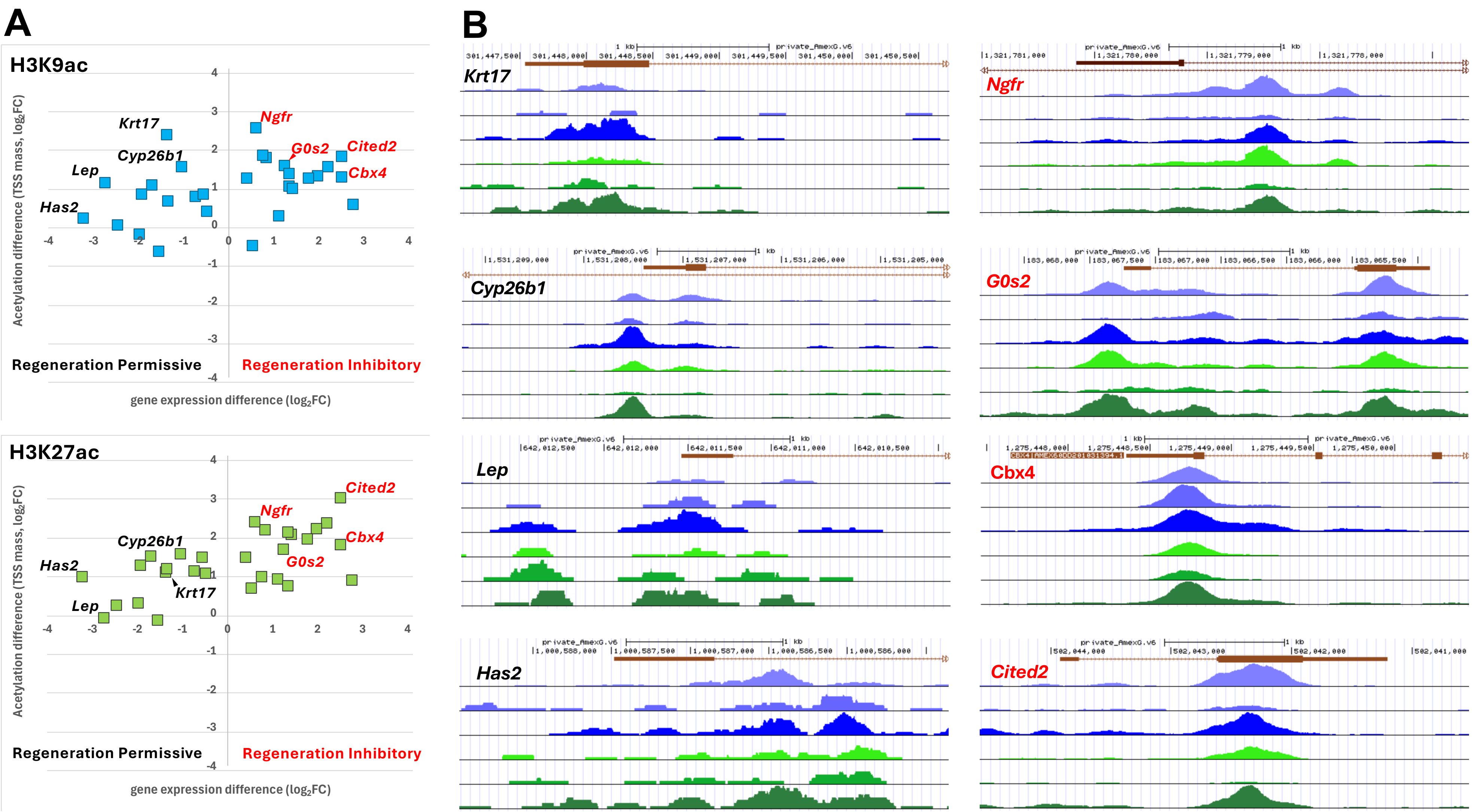

Fig. S3. Relationship between promoter acetylation and transcription in response to romidepsin treatment for regeneration permissive and inhibitory genes. A) Plots showing H3K9ac and H3K27ac for promoters relative to log_2 fold change between control and romidepsin-treated embryos at 6-HPA. Gene expression values are from Voss et al 2021. B) Genome browser images showing H3K9ac and H3K27ac data tracks for select regeneration permissive (*Krt17, Cyp26b1, Lep, Has2*) and inhibitory (*Ngfr, G0s2, Cbx4, Cited2*) genes.

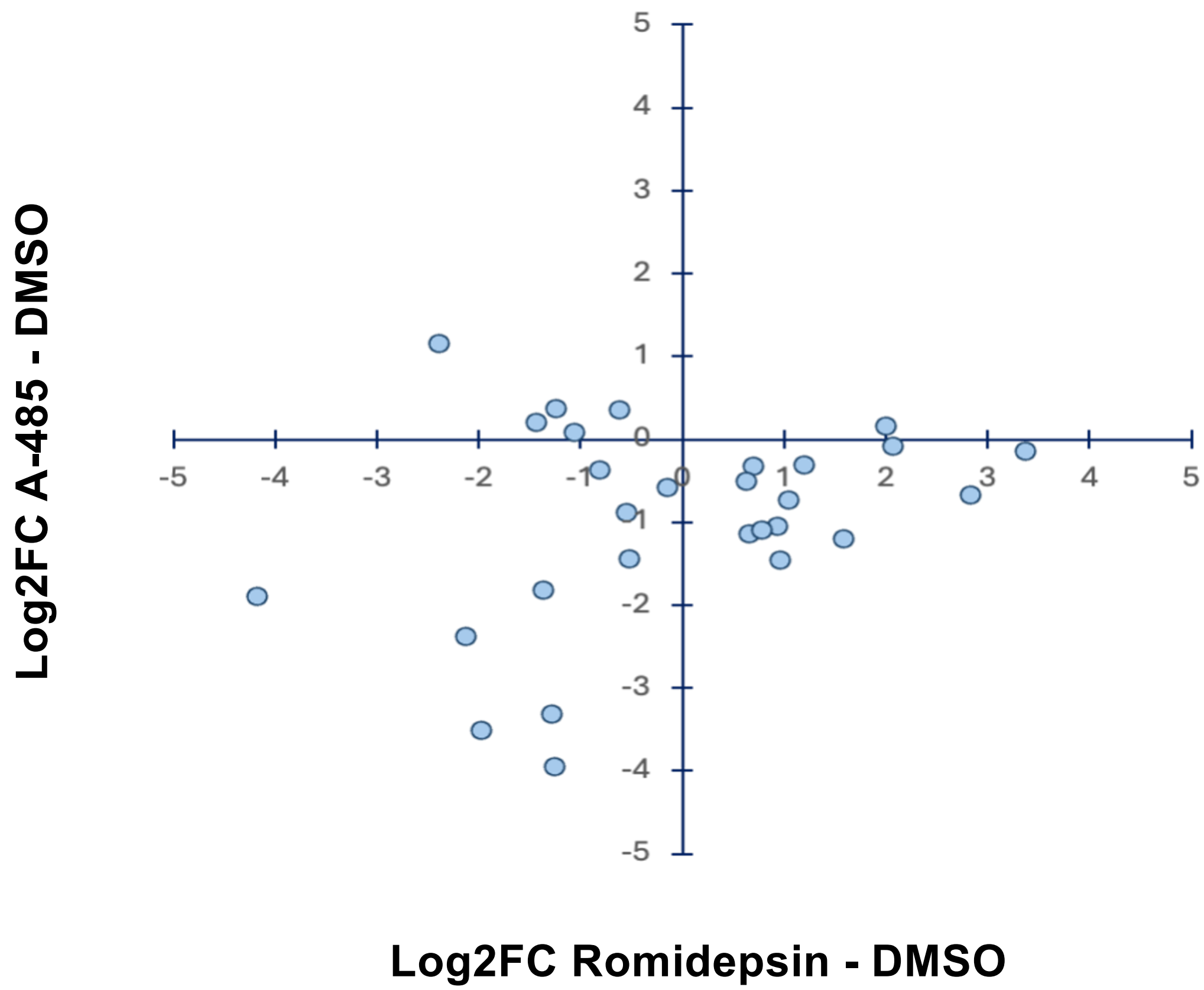

Fig. S4. Effect of romidepsin and A-485 on expression of 28 regeneration permissive and inhibitory genes. The romidepsin values were obtained from Table 2 in Voss et al (2019).

Biology Open • Supplementary information

Table S1. Summary statistics for ChIP-seq data.

Available for download at
https://journals.biologists.com/bio/article-lookup/doi/10.1242/bio.062726#supplementary-data

Table S2. Changes in gene expression and promoter acetylation for all expressed genes with evidence of acetylation (promoter mass > 200 in at least one sample). Bivariate rank data for genes differentially expressed between C-6HPA and R-6HPA (p-value < 0.1).

Available for download at
https://journals.biologists.com/bio/article-lookup/doi/10.1242/bio.062726#supplementary-data

Table S3. Panther protein classifications that were significantly enriched by genes with the highest (top 5%) bivariate ranking scores for promoter acetylation and gene expression.

Available for download at
https://journals.biologists.com/bio/article-lookup/doi/10.1242/bio.062726#supplementary-data

Table S4. Differentially acetylated proteins identified between contrasts of 0-HPA, C-6HPA, and R-6HPA samples.

Available for download at
https://journals.biologists.com/bio/article-lookup/doi/10.1242/bio.062726#supplementary-data

Table S5. Biological process ontologies that were significantly enriched by differentially acetylated proteins identified between contrasts of 0-HPA, C-6HPA, and R-6HPA samples.

Available for download at
https://journals.biologists.com/bio/article-lookup/doi/10.1242/bio.062726#supplementary-data

Table S6. Differentially expressed genes identified between DMSO and A-485 treated embryos at 24 HPA.

Available for download at
https://journals.biologists.com/bio/article-lookup/doi/10.1242/bio.062726#supplementary-data

Table S7. Biological process ontologies that were significantly enriched by the list of genes that were significantly differentially expressed between DMSO and A-485 treated embryos at 24 HPA.

Available for download at
https://journals.biologists.com/bio/article-lookup/doi/10.1242/bio.062726#supplementary-data
