# Supplementary material for: Histone and non-histone protein acetylation in a tail regeneration model (Ambystoma mexicanum)
Source: Biol Open. 2026 Jul 14;15(7):bio062726. doi: 10.1242/bio.062726 (PMC13446560; doi:10.1242/bio.062726)
Supplement: Supplementary file 2 [file biolopen-15-062726_review_history.pdf]

Histone and Non-Histone Protein Acetylation in a Tail Regeneration Model (Ambystoma mexicanum)

Nataliya Timoshevskaya, Raissa Cecil, James Schwartz, Jeramiah Smith and Stephen Randal Voss

10.1242/bio.062726

Editor: Tristan Rodríguez

Review timeline

Submission to sister journal:	12 January 2026
Editorial decision at sister journal:	6 March 2026
Resubmission at sister journal:	20 April 2026
Editorial decision:	25 April 2026
Transfer to Biology Open:	1 June 2026
Accepted:	5 June 2026

Original submission to sister journal

<u>First decision letter</u>

MS Title: Histone and Non-Histone Protein Acetylation in a Tail Regeneration Model (Ambystoma mexicanum)

Authors: Nataliya Timoshevskaya; Raissa Cecil; James Schwartz; Jeramiah Smith; Stephen Randal Voss

I have now received all the referees reports on the above manuscript and have reached a decision. The referees' comments are appended below.

As you will see from their reports, the referees recognise the potential of your work, but they also raise significant concerns about it. Given the nature of these concerns, I am afraid I have little choice other than to reject the paper at this stage.

However, having evaluated the paper, I do recognise the potential importance of this work. I would therefore be prepared to consider as a new submission an extension of this study that contains new experiments, data and discussions and that address fully the major concerns of the referees. The work required goes beyond a standard revision of the paper. Please bear in mind that the referees (who may be different from the present reviewers) will assess the novelty of your work in the context of all previous publications, including those published between now and the time of resubmission. There is interest but the reviews are mixed and I would understand if you chose to submit elsewhere. In particular, Reviewers 1 and 3 have several concerns including mechanistic questions expected to be addressed in the study that are not answered. If you were to submit a manuscript with additions you feel address the key issues, I would likely try to send this back to the 3 reviewers and consult a 4th.

If you decide to resubmit, please go to: https://www.editorialmanager.com/develop/ and click on the 'Submit a manuscript' link.

Please ensure that you indicate that this is a resubmission, and enter your manuscript identification number (dev.205503). I would also ask you to provide in the cover letter an explanation of the key

© 2026. Published by The Company of Biologists under the terms of the Creative Commons Attribution License (https://creativecommons.org/licenses/by/4.0/).

ways in which the manuscript differs from the current submission, followed by a point-by-point response to the referees' concerns.

We do understand that the work entailed in a potential new submission is significant, and that you may prefer to submit elsewhere without further delay.

Reviewer 1: SUMMARY OF THE ADVANCE MADE IN THIS PAPER AND ITS POTENTIAL SIGNIFICANCE TO THE FIELD

This manuscript builds on the authors' 2019 and 2021 studies, which showed that administration of romidepsin, a Class I Histone deacetylase (HDAC) inhibitor, impairs Axolotl tail regeneration. This manuscript further performs proteomics and epigenomic analyses to investigate histone and non-histone acetylation changes and potential target effectors of the HDAC inhibitor. Using a large number of samples, the authors conducted mass spectrometry, ChIP-seq, and CUT&RUN approaches and identified global changes in acetylation levels of various histones in response to HDAC inhibitor treatment. The authors found that genes deacetylated upon injury are likely susceptible to HDAC inhibitor-mediated hyperacetylation. However, when compared with regeneration permissive and inhibitory genes defined in previous transcriptomic analyses (Voss et al., 2021), the authors found that histone acetylation appears to correlate poorly with injury-induced or injury-reduced gene expression. Nano LC-MS/MS approaches establish valuable proteome profiles to characterize acetylated proteins following HDAC inhibitor treatment during regeneration. The authors focused on Ep300/Crebbp as their acetylation levels were altered by HDAC inhibitor treatment. Pharmacological inhibition of Crebbp/Ep300 demonstrates the importance of Crebbp/Ep300 on tail regeneration.

SUGGESTIONS TO AUTHORS

Overall, the authors generate valuable epigenomic and proteomic datasets. However, several major key claims remain preliminary. The link between histone acetylation and regeneration-associated gene expression is unclear. Evidence supporting non-histone targets, such as Crebbp/Ep300, by HDACi-related acetylation is insufficient. Moreover, the functional consequences of these modifications during regeneration remain unresolved. Additional experimental validation and mechanistic analyses are necessary to substantiate the conclusions.

I have several concerns outlined below:

1. Link between acetylation and transcription change

The connection between HDACi-mediated histone acetylation change and transcriptional regulation is not clarified. More comprehensive integration of transcriptomic, epigenomic, and proteomic analyses is needed. The authors assessed H3K9ac and H3K27ac levels at the promoters of small number of genes determined from previous studies. More genome-wide transcriptome and epigenome analyses should be performed to understand the impact of H3K9ac and H3K27ac on expression of injury-inducible and injury-reduced gene expression. The current data are insufficient to establish a meaningful relationship between histone acetylation and transcriptional regulation.

2. Proteomic data - Nano LC-MS/MS

The authors compared acetylated peptides analyzed by Nano LC-MS/MS. However, more informative approach would be comparing total or non-acetylated peptide vs acetylated peptide within the same samples. The most abundantly detected peptides are H3.3 and H2A.Z in 0 HPA vs 6 HPA DMOS and 0 HPA vs 6 HPA Romi. But, their expression levels are not apparent in 6 HPA DMOS vs 6 HPA Romi. Regarding EP300, multiple acetylated peptides (XP_069510525_1_K79, XP_069510525_1_K1807 and XP_069510525_1_K1803) are detectable. These data suggest that comparing peptide levels representing total Ep300 abundance and acetylated Ep300 could be feasible. In addition, Ep300 (XP_069510525_1_K79) and Crebp (XP_069502924_1_K73) acetylated peptide levels are less than 1.5 value. By contrast, value for EP300 other peptides (XP_069510525_1_K1807 and XP_069510525_1_K1803) are detectable with higher level (over 6). What is the biological implication of this value? Are these values within a meaningful detection

© 2026. Published by The Company of Biologists under the terms of the Creative Commons Attribution License (https://creativecommons.org/licenses/by/4.0/).

range? Are there other validations confirming that these lysine residues are acetylated upon injury and reversed by HDAC inhibitor treatment? Further validation is needed. Although technically challenging, supporting experiments such as biochemical assays (wild-type versus lysine-mutant proteins incubated with HDACs or HATs) or genetic approaches (expression of wild-type and mutant proteins followed by regeneration assays) would greatly strengthen the conclusions.

3. Validation of histone modification level

In addition to Mass Spectrometry, the authors require to validate histone modification change by western blot and/or immunohistochemistry (IHC). In particular, IHC could provide valuable insight into cell-type-specific regulation of histone modifications during regeneration.

4. Statements such as: "How histone deacetylase inhibitors (HDACi) alter protein acetylation to potently inhibit tissue regeneration is currently unknown" and "To our knowledge, no study has characterized HDACi-mediated effects on histone and nonhistone protein acetylation during tissue regeneration, changes that hypothetically may associate with transcriptional regulation and regeneration outcome.", are overly strong. A substantial body of literature has already investigated HDAC inhibition in tissue regeneration contexts. These statements should be appropriately moderated to reflect existing work in the field.

Heart: https://doi.org/10.3390/jcm13247797, https://doi.org/10.1371/journal.pgen.1009890
Wound healing and skin: DOI:10.1126/scitranslmed.ads0594, https://doi.org/10.1111/exd.70060
Liver: https://doi.org/10.1016/j.molcel.2016.05.029
Bone: https://doi.org/10.1186/s13287-025-04719-2

Reviewer 2: SUMMARY OF THE ADVANCE MADE IN THIS PAPER AND ITS POTENTIAL SIGNIFICANCE TO THE FIELD

How epigenetic changes govern regeneration of complex body parts remains an under-explored aspect of currently published research, particularly in salamander models. There is a knowledge gap around why and how HDAC inhibitors impair regeneration, for example, what are the target genes relevant for specific aspects of regeneration? This group previously reported that histone deacetylase inhibitors can interfere with axolotl tail regeneration, bu the concrete mechanisms for this relationship remained unclear. In this work, the authors used unbiased and "brute-force" approaches to globally characterize changes to the epigenome that may underlie the dependency of axolotl tail regeneration on precise regulation of histone acetylation, a key regulator of local genome accessibility. They specifically assayed H3K9ac and H3K27ac across the genome. They used these data to make broad statements about how acetylation, and therefore likely chromatin accessibility, is altered within genomic regions where genes are typically upregulated or downregulated during the early phases of axolotl tail regeneration. Overall, there was not a simplistic story to emerge, but that's okay (and it's the biology). What's exciting here is that these authors were thorough and exhaustive, and they've provided the field with a wealth of high-quality data that everyone can use. To help bolster the validity of their analyses, they used a pharmacological inhibitor of one identified candidate target and showed this inhibited tail regeneration. So, there is a morsel of "experimental validation" here as well.

SUGGESTIONS TO AUTHORS

Even though the authors were careful to not over-interpret their findings, binning the genes identified as simply "regeneration permissive" and "regeneration inhibitory" is still a very coarse way of expressing the findings. However, they do have to express the relationship of what they found to the existing transcriptomic literature, so perhaps that is the safest. The authors clearly know that an individual gene or protein's activity could at one time point, or in one cell type, be said to "promote" or "inhibit" regeneration (even p53 activity is one example), and they do address that in the text as well as in the end, in the limitations section. However, it might be better to frame this whole study overall as a "RESOURCE" rather than a research article. I think that would set the expectation for readers but also really emphasize the high quality of the data and the power it holds to inform many different studies in the future. This is a real tour-de-force (some samples required 1200 specimens!) and I don't think we should hold the authors to really dig in and follow

© 2026. Published by The Company of Biologists under the terms of the Creative Commons Attribution License (https://creativecommons.org/licenses/by/4.0/).

up "mechanistically" on any of the exact loci implicated in this analysis; that would hold up scientific progress.

A couple of very minor issues I wondered about:

1) In the section entitled, "Proteome-level effects of romidepsin on lysine acetylation"--The verbiage "contrast" is clunky and I found it confusing--do they mean "comparison?"

2) The terminology for the stage of axolotls they used--From the pictures in the figures and the methods, wouldn't it make more sense to call those "hatchlings" or "larvae" rather than embryos??

Reviewer 3: SUMMARY OF THE ADVANCE MADE IN THIS PAPER AND ITS POTENTIAL SIGNIFICANCE TO THE FIELD

For over a decade, it has been recognized that treatment with histone deacetylase inhibitors (HDACi) leads to attenuation of regeneration responses in amphibians. This study by Timoshevskaya and colleagues seeks to discern the mechanism of this effect by directly interrogating how acetylation levels in histones and in other proteins are changed by treatment with Romidepsin, an HDACi that this group has previously studied. This is important work because the phenomenon of HDACi effects on regeneration has been presumed to derive from an overall increase in chromatin acetylation, accessibility, and transcription, but these relationships have not previously been directly shown. The effects of HDACi on non-histone protein acetylation have also not been previously catalogued in a regeneration context— equally important as these may instead, or also, be responsible for the regeneration inhibition that is observed with these drugs. The authors find that Romidepsin treatment does indeed have the expected effect, raising global levels of acetylation in many histone lysine residues (by mass spec), and increasing the number of peaks for lysines commonly associated with increased transcription (H3K9ac and H3K27ac)(by ChIP-Seq and CUT&RUN). Effects on transcription were less coherent, with some genes both up- and down-regulated. To assess the effects of regeneration and Romidepsin treatment on non-histone acetylation, they profiled acetylated peptides across the proteome, finding vast changes in acetylation with regeneration. Many peptides were also altered in acetylation with Romidepsin. CREBBP was particularly noted as a Romidepsin-sensitive acetylated protein. Pharmacological inhibition of CREBBP by A-485 also inhibited regeneration and led to transcriptional changes, including some genes that are also regulated by Romidepsin.
My overall impression is that this study provides both some important confirmatory and some surprising findings about the overall effects of using a general class I HDACi in the context of amphibian regeneration. But it leaves most potential mechanistic questions unanswered: it's still unclear whether the failure to regenerate after treatment derives from effects on histones, histone-independent effects on wound healing, effects on transcription independent of histone or non-histone acetylation, or all of the above. And while it presents the interesting and paradoxical findings that inhibiting HDACs increases histone acetylation while sometimes decreasing transcription, and that it leads to increased acetylation at many peptides but decreased acetylation at others, it doesn't really uncover how. The consequences of Romidepsin treatment are presented in a more list-like fashion than with a mechanistic narrative to understand them. In the present form, I think the study would make more sense for a different journal, but if one or more of the above issues can be more meaningfully addressed it would make it more suited for this journal. I do think the work makes an important contribution, and would be happy to read a revised version.

SUGGESTIONS TO AUTHORS

Essential revisions and problems that need to be addressed:

1. The A485 studies are not well controlled. Given that a theme of the paper is that pharmacological inhibitors can have broader effects than intended, it stands out that neither efficacy nor specificity of A485 is demonstrated here. I think the most straightforward way to address this would be to treat larvae with A485, Romidepsin, or DMSO, and use mass spec to assay the acetylation level of CREBBP peptides, as the intended target of A485, as well as at least a couple other proteins that are strongly differentially acetylated by Romidepsin but not expected to be targets of A485. Loss of acetylation of CREBBP but not other Romidepsin-senisitve peptides

© 2026. Published by The Company of Biologists under the terms of the Creative Commons Attribution License (https://creativecommons.org/licenses/by/4.0/).

would show both efficacy and specificity. Another strategy would be to use a different, orthogonal pharmacological inhibitor of CREBBP (if available) and show the same effects. Even better would be a mutant version of CREBBP that could act as a dominant negative (missing key acetylation residues or even a catalytically impaired mutant, as in PMID: 29551561), but I'm not sure if that is feasible in this axolotl regeneration context.

2. The read depth for ChIP-SEQ data needs context, since the axolotl genome is huge. 11-30 million reads may not be deep enough for reproducible peak calling for these very abundant marks. (For the human genome, one would typically shoot for about 30 million reads for these marks, and that genome is 1/10th the axolotl's size). I think it's okay to create a union set, but what is the level of concordance in peak calls between replicates? That will give a sense of whether the sequencing is deep enough/well-powered enough. Some Venn diagrams and statistical analysis would help here. Also, could the authors please comment further in the methods on how data were normalized to ensure that fewer/more peaks weren't just called because of read depth—were libraries downsampled to a uniform number of reads and compared, for example? Normalized by FPKM or similar? I know these are fairly standard processing steps but it would be helpful to be able to see how they were applied.

3. The discordance between increased acetylation and increased transcription is very interesting, because it unseats our general expectation of how transcriptional regulation works. To me, there are two ways one should test the relationship between acetylation and transcription after Romidepsin. One is to ask if transcriptionally upregulated genes also have increased acetylation. This the authors looked at (at least for 29 genes), and seems to be generally but not always true (Supp Fig 3). But it's at least equally important to ask the question the other way, which I don't think was done: do genes with increased acetylation (in promoters or gene bodies) have increased transcription? So when the authors say "Gene ontology (GO) enrichment analyses of genes associated with romidepsin-sensitive promoters (red dots in Figure 3) identified a variety of enriched developmental processes and cell signaling pathways, including TGF, WNT, BMP, NOTCH, EGFR, PDGF, VGF, and ERBB (Supplementary Table 2)"—are these genes transcriptionally increased, decreased, or unchanged relative to DMSO treatment at the same timepoints?

4. I didn't really follow what the authors make of the decreases in acetylation that are observed for non-histones like the clotting factors after Romidepsin treatment. What hypotheses may explain these observations? More specifically, I think the mass spec measurements would allow them to parse a loss of protein abundance (all peptides for that protein decrease, explainable by decreased transcription or translation, or increased degradation) versus a loss of acetylation (the ratio of acetylated to non-acetylated peptides for that protein decreased, explainable by loss of a non-histone acetyltransferase or gain of a deacetylase). For which of the non-histone proteins that have decreased acetylated peptides are they seeing a loss of protein, and for which are they seeing a loss of acetylation? Clarifying this would help the reader better understand whether these are changes that might be caused by decreases in transcription, such as by CREBBP. I think it would be helpful if some of the non-histone data appeared in a main figure, rather than just a supplemental table, which was a bit hard to parse.

5. Romidepsin treatment was previously noted as remarkable by this group because it led to altered regeneration outcomes after just 1 minute of treatment, which they stress again here. But the effects assayed are all after 6 or 24h. Does Romidepsin treatment for 1 minute change acetylation of any of these targets? Maybe the authors could assay a few of the marks in Figure 1 that were the strongest responders after 6h to see if those also respond to a 1 minute treatment. If they don't respond, then the effects of Romidepsin on regeneration may have more to do with clotting proteins and immediate wound response than transcription?

Minor comments and suggestions:

1. Standard capitalization conventions should be followed: CUT&RUN, ChIP-SEQ

2. I think a schematic Figure 1 that laid out how the experiment was done and how the data were analyzed might make the overall workflow and types of conclusions drawn easier to follow.

© 2026. Published by The Company of Biologists under the terms of the Creative Commons Attribution License (https://creativecommons.org/licenses/by/4.0/).

3. The parallels drawn between regeneration and cancer in the Introduction (and a bit elsewhere, too) feel a little too broad or forced. Tumor suppressors and oncogenes have specific definitions. Not every inflammation-promoting factor should be equated to a tumor suppressor.

3. Could the authors add a bit more comment about whether/how some of their observations may be influenced by tissue- or cell type-specific effects? For example, could gains of acetylation in one cell type cause a local increase in transcription that's being swamped out by relatively low abundance of that cell type, or losses of transcription in a different cell type?

4. It is just generally always hard to keep manuscripts about lists of genes, directionality of regulation, and lists of GO terms clear and easy to follow. Can't be prevented. I do think the authors could help their readers out by going over the Results and Discussion once more. It would help to break paragraphs into smaller ideas with clear topic sentences and clear conclusion sentences, and make sure that the logic in each sentence and paragraph follows easily from observation to conclusion. (Sorry for this—I have written plenty of mushy paragraphs about GO terms myself, and I know it is challenging to fix).

Resubmission to sister journal

First decision letter

MS Title: Histone and Non-Histone Protein Acetylation in a Tail Regeneration Model (Ambystoma mexicanum)

Authors: Nataliya Timoshevskaya; Raissa Cecil; James Schwartz; Jeramiah Smith; Stephen Randal Voss

Many thanks for submitting your manuscript to us. I have assessed it and consulted one of the previous reviewers. My view is that, while the profiles are potentially valuable for the axolotl regeneration community, there would be significant requests by reviewers for experiments that clarify potential conclusions from the profiles and pharmacologic perturbation. I hope that this rapid decision will give you the opportunity to submit your work elsewhere without further delay.

Author response to reviewers' comments

Reviewer 1: SUMMARY OF THE ADVANCE MADE IN THIS PAPER AND ITS POTENTIAL SIGNIFICANCE TO THE FIELD

This manuscript builds on the authors' 2019 and 2021 studies, which showed that administration of romidepsin, a Class I Histone deacetylase (HDAC) inhibitor, impairs Axolotl tail regeneration. This manuscript further performs proteomics and epigenomic analyses to investigate histone and non-histone acetylation changes and potential target effectors of the HDAC inhibitor. Using a large number of samples, the authors conducted mass spectrometry, ChIP-seq, and CUT&RUN approaches and identified global changes in acetylation levels of various histones in response to HDAC inhibitor treatment. The authors found that genes deacetylated upon injury are likely susceptible to HDAC inhibitor-mediated hyperacetylation. However, when compared with regeneration permissive and inhibitory genes defined in previous transcriptomic analyses (Voss et al., 2021), the authors found that histone acetylation appears to correlate poorly with injury-induced or injury-reduced gene expression. Nano LC-MS/MS approaches establish valuable proteome profiles to characterize acetylated proteins following HDAC inhibitor treatment during regeneration. The authors focused on Ep300/Crebbp as their acetylation levels were altered by HDAC inhibitor treatment. Pharmacological inhibition of Crebbp/Ep300 demonstrates the importance of Crebbp/Ep300 on tail regeneration.

SUGGESTIONS TO AUTHORS

© 2026. Published by The Company of Biologists under the terms of the Creative Commons Attribution License (https://creativecommons.org/licenses/by/4.0/).

Overall, the authors generate valuable epigenomic and proteomic datasets. However, several major key claims remain preliminary. The link between histone acetylation and regeneration-associated gene expression is unclear. Evidence supporting non-histone targets, such as Crebbp/Ep300, by HDACi-related acetylation is insufficient. Moreover, the functional consequences of these modifications during regeneration remain unresolved. Additional experimental validation and mechanistic analyses are necessary to substantiate the conclusions.

We appreciate the opportunity to improve our manuscript and address your concerns below, which have greatly improved our manuscript and the significance of our findings. We rewrote the abstract to better highlight these findings, rewrote a paragraph in the introduction, added a new section in the results and rewrote some of the original paragraphs, and rewrote most of the discussion. We added new figures, tables, and references. Overall, the manuscript is greatly improved.

1. Link between acetylation and transcription change
The connection between HDACi-mediated histone acetylation change and transcriptional regulation is not clarified. More comprehensive integration of transcriptomic, epigenomic, and proteomic analyses is needed. The authors assessed H3K9ac and H3K27ac levels at the promoters of small number of genes determined from previous studies. More genome-wide transcriptome and epigenome analyses should be performed to understand the impact of H3K9ac and H3K27ac on expression of injury-inducible and injury-reduced gene expression. The current data are insufficient to establish a meaningful relationship between histone acetylation and transcriptional regulation.

We agree, while the original manuscript established that romidepsin globally increases acetylation, we now recognize that it did not sufficiently explore the relationship between acetylation and gene expression. In the revision, we report results of additional analyses that investigate the relationship between histone acetylation and gene expression. Details of these analyses and results are included in the results section of the revised manuscript under the subheading: *Relationship Between Histone Acetylation and Gene Expression.* These new analyses show that the effect of romidepsin on histone acetylation is global but more nuanced for gene expression. We examined the relationship between acetylation and gene expression for all genes with evidence of promoter acetylation and found that these two variables are only weakly associated, similar to reports in the cancer field. However, when we looked specifically at genes that are significantly differentially expressed between control and romidepsin samples, we found that the magnitude of promoter acetylation was significantly increased for genes that were up-regulated versus down-regulated by romidepsin. This is a robust finding; we obtained this result when independently analyzing thousands of H3K9ac and H3K27ac promoters, and when analyzing the N=29 regeneration permissive and inhibitory gene set that we highlighted in the original manuscript. Furthermore, we found that transcription factors and regeneration inhibitory genes were enriched among genes that were most highly acetylated and expressed in response to romidepsin treatment. Overall, these results strongly implicate romidepsin mediated promoter acetylation in the expression of regeneration inhibitory genes. We include 3 new figures (Figures 4-6) and 2 new supplementary tables (Supplementary Table 2,3) to detail these new results; also, original Figure 4 is now Supplementary Figure 3 in the revised manuscript.

2. Proteomic data - Nano LC-MS/MS
The authors compared acetylated peptides analyzed by Nano LC-MS/MS. However, more informative approach would be comparing total or non-acetylated peptide vs acetylated peptide within the same samples. The most abundantly detected peptides are H3.3 and H2A.Z in 0 HPA vs 6 HPA DMOS and 0 HPA vs 6 HPA Romi. But, their expression levels are not apparent in 6 HPA DMOS vs 6 HPA Romi. Regarding EP300, multiple acetylated peptides (XP_069510525_1_K79, XP_069510525_1_K1807 and XP_069510525_1_K1803) are detectable. These data suggest that comparing peptide levels representing total Ep300 abundance and acetylated Ep300 could be feasible. In addition, Ep300 (XP_069510525_1_K79) and Crebp (XP_069502924_1_K73) acetylated peptide levels are less than 1.5 value. By contrast, value for EP300 other peptides (XP_069510525_1_K1807 and XP_069510525_1_K1803) are detectable with higher level (over 6). What is the biological implication of this value? Are these values within a meaningful detection range? Are there other validations confirming that these lysine residues are acetylated upon injury

© 2026. Published by The Company of Biologists under the terms of the Creative Commons Attribution License (https://creativecommons.org/licenses/by/4.0/).

and reversed by HDAC inhibitor treatment? Further validation is needed. Although technically challenging, supporting experiments such as biochemical assays (wild-type versus lysine-mutant proteins incubated with HDACs or HATs) or genetic approaches (expression of wild-type and mutant proteins followed by regeneration assays) would greatly strengthen the conclusions.

We agree that it is important to understand the stoichiometry of lysine acetylation. It seems likely that transcriptional regulation will depend upon the fraction of acetylated peptides in a protein and not simply the presence or absence of acetylation, and it seems likely that the functions of lowly and highly abundant protein may be associated with stoichiometry. Recent progress is being made to achieve unbiased analyses of acetylation stoichiometry *in vitro* with cell lines, but it would currently be difficult if not impossible to quantify the relative abundances of native and acetylated peptides, and total protein abundances, from our limiting embryo tail samples. Looking back over the last 15 years, studies have primarily used anti-acetyl lysine antibodies to enrich peptides because this is the best way to comprehensively sample and characterize lowly abundant, acetylated peptides, which was our goal in this inaugural analysis of non-histone protein acetylation in the axolotl. Characterization of more than one peptide for Ep300 after acetyl antibody enrichment doesn't necessarily mean that these same peptides would be identified in a full proteomic screen without enrichment. It is generally known that the ability to detect acetylated peptides depends upon protein abundance, and that low abundance peptides are difficult to detect without enrichment. This is a general limitation of MS/MS studies. The biological implication of peptide abundance is context specific, reflecting MS/MS resolving power and relative protein abundance, which varies across proteins. The values that we report in the supplementary table are normalized relative peptide abundances; we add this label to the table in the revised manuscript. We have added an additional sentence in the discussion to acknowledge limitations of the Nano LC-MS/MS approach and the need to work towards analyses that can reveal the stoichiometry of lysine acetylation during regeneration.

The reason H3.3 and H2A.Z were not reported as differentially acetylated between 6 HPA DMSO and 6 HPA Romi is because both samples showed a similar increase in acetylation relative to 0 HPA DMSO.

We highlighted results showing increased acetylation of hemostasis proteins across independent samples because it is biologically interesting and because it showed replicability of MS/MS results, which validated the approach we used.

We agree that it is important to test specific lysine sites for function. However, it would be difficult to engineer lysine acetylation modifications in proteins for functional tests in the axolotl. We highlight this in the discussion section under 'Caveats' and point out that differentially acetylated lysines that we identified for Runx1 were validated for function by changing these residues to arginine in an in vitro model.

3. Validation of histone modification level
In addition to Mass Spectrometry, the authors require to validate histone modification change by western blot and/or immunohistochemistry (IHC). In particular, IHC could provide valuable insight into cell-type-specific regulation of histone modifications during regeneration.

We validated romidepsin-mediated histone acetylation using two additional approaches: ChiP-SEQ and CUT&RUN. IHC is a low-resolution qualitative approach; using an antibody at a cellular level would not address differential acetylation of specific histone or non-histone lysine residues. Acetylation marks are detectable in all samples, and we would expect positive IHC signal in all cells. Our targeted mass spec analysis of histones (Figure 2) is in line with these expectations, and we might consider it to be essentially a higher resolution version of the proposed western blot study. We think it clear that the gene-level distribution of acetylation marks changes during early regeneration and is modulated by inhibition of class 1 HDACs, and don't want to give the impression that acetylation is completely lost during regeneration.

4. Statements such as: "How histone deacetylase inhibitors (HDACi) alter protein acetylation to potently inhibit tissue regeneration is currently unknown" and "To our knowledge, no study has characterized HDACi-mediated effects on histone and nonhistone protein acetylation during tissue

© 2026. Published by The Company of Biologists under the terms of the Creative Commons Attribution License (https://creativecommons.org/licenses/by/4.0/).

regeneration, changes that hypothetically may associate with transcriptional regulation and regeneration outcome.", are overly strong. A substantial body of literature has already investigated HDAC inhibition in tissue regeneration contexts. These statements should be appropriately moderated to reflect existing work in the field.

Heart: https://doi.org/10.3390/jcm13247797, https://doi.org/10.1371/journal.pgen.1009890
Wound healing and skin: DOI:10.1126/scitranslmed.ads0594, https://doi.org/10.1111/exd.70060
Liver: https://doi.org/10.1016/j.molcel.2016.05.029
Bone: https://doi.org/10.1186/s13287-025-04719-2

We agree, a substantial body of literature has investigated HDAC inhibition in different contexts and documented functional changes at organismal, cellular, and physiological levels. The listed papers primarily address wound healing. Relatively few studies of regeneration, not just wound-healing, have administered HDACi and identified molecular-level changes, specifically, histone and non-histone acetylation changes in the same study. We toned down the first sentence by replacing "currently unknown" to "relatively unexplored". We toned down the second sentence by deleting "To our knowledge" and replacing it with "Few studies have characterized…."

Reviewer 2: SUMMARY OF THE ADVANCE MADE IN THIS PAPER AND ITS POTENTIAL SIGNIFICANCE TO THE FIELD

How epigenetic changes govern regeneration of complex body parts remains an under-explored aspect of currently published research, particularly in salamander models. There is a knowledge gap around why and how HDAC inhibitors impair regeneration, for example, what are the target genes relevant for specific aspects of regeneration? This group previously reported that histone deacetylase inhibitors can interfere with axolotl tail regeneration, bu the concrete mechanisms for this relationship remained unclear. In this work, the authors used unbiased and "brute-force" approaches to globally characterize changes to the epigenome that may underlie the dependency of axolotl tail regeneration on precise regulation of histone acetylation, a key regulator of local genome accessibility. They specifically assayed H3K9ac and H3K27ac across the genome. They used these data to make broad statements about how acetylation, and therefore likely chromatin accessibility, is altered within genomic regions where genes are typically upregulated or downregulated during the early phases of axolotl tail regeneration. Overall, there was not a simplistic story to emerge, but that's okay (and it's the biology). What's exciting here is that these authors were thorough and exhaustive, and they've provided the field with a wealth of high-quality data that everyone can use. To help bolster the validity of their analyses, they used a pharmacological inhibitor of one identified candidate target and showed this inhibited tail regeneration. So, there is a morsel of "experimental validation" here as well.

SUGGESTIONS TO AUTHORS

Even though the authors were careful to not over-interpret their findings, binning the genes identified as simply "regeneration permissive" and "regeneration inhibitory" is still a very coarse way of expressing the findings. However, they do have to express the relationship of what they found to the existing transcriptomic literature, so perhaps that is the safest. The authors clearly know that an individual gene or protein's activity could at one time point, or in one cell type, be said to "promote" or "inhibit" regeneration (even p53 activity is one example), and they do address that in the text as well as in the end, in the limitations section. However, it might be better to frame this whole study overall as a "RESOURCE" rather than a research article. I think that would set the expectation for readers but also really emphasize the high quality of the data and the power it holds to inform many different studies in the future. This is a real tour-de-force (some samples required 1200 specimens!) and I don't think we should hold the authors to really dig in and follow up "mechanistically" on any of the exact loci implicated in this analysis; that would hold up scientific progress.

We appreciate the reviewer's comments and insight, and recognition of the value of our findings to the regeneration community. Our study does establish Romidepsin as a positive control for future acetylation studies in the axolotl and we highlight this point in the discussion as it is a significant finding of our work.

© 2026. Published by The Company of Biologists under the terms of the Creative Commons Attribution License (https://creativecommons.org/licenses/by/4.0/).

A couple of very minor issues I wondered about:
1) In the section entitled, "Proteome-level effects of romidepsin on lysine acetylation"--The verbiage "contrast" is clunky and I found it confusing--do they mean "comparison?"

Yes, we mean comparison and changed this wording.

2) The terminology for the stage of axolotls they used--From the pictures in the figures and the methods, wouldn't it make more sense to call those "hatchlings" or "larvae" rather than embryos??

Regarding hatchlings vs larvae, at University of Kentucky they are embryos until they become free-living and feed (we used pre-hatch embryos for the assay).

Reviewer 3: SUMMARY OF THE ADVANCE MADE IN THIS PAPER AND ITS POTENTIAL SIGNIFICANCE TO THE FIELD

For over a decade, it has been recognized that treatment with histone deacetylase inhibitors (HDACi) leads to attenuation of regeneration responses in amphibians. This study by Timoshevskaya and colleagues seeks to discern the mechanism of this effect by directly interrogating how acetylation levels in histones and in other proteins are changed by treatment with Romidepsin, an HDACi that this group has previously studied. This is important work because the phenomenon of HDACi effects on regeneration has been presumed to derive from an overall increase in chromatin acetylation, accessibility, and transcription, but these relationships have not previously been directly shown. The effects of HDACi on non-histone protein acetylation have also not been previously catalogued in a regeneration context— equally important as these may instead, or also, be responsible for the regeneration inhibition that is observed with these drugs. The authors find that Romidepsin treatment does indeed have the expected effect, raising global levels of acetylation in many histone lysine residues (by mass spec), and increasing the number of peaks for lysines commonly associated with increased transcription (H3K9ac and H3K27ac)(by ChIP-Seq and CUT&RUN). Effects on transcription were less coherent, with some genes both up- and down-regulated. To assess the effects of regeneration and Romidepsin treatment on non-histone acetylation, they profiled acetylated peptides across the proteome, finding vast changes in acetylation with regeneration. Many peptides were also altered in acetylation with Romidepsin. CREBBP was particularly noted as a Romidepsin-sensitive acetylated protein. Pharmacological inhibition of CREBBP by A-485 also inhibited regeneration and led to transcriptional changes, including some genes that are also regulated by Romidepsin.

My overall impression is that this study provides both some important confirmatory and some surprising findings about the overall effects of using a general class I HDACi in the context of amphibian regeneration. But it leaves most potential mechanistic questions unanswered: it's still unclear whether the failure to regenerate after treatment derives from effects on histones, histone-independent effects on wound healing, effects on transcription independent of histone or non-histone acetylation, or all of the above. And while it presents the interesting and paradoxical findings that inhibiting HDACs increases histone acetylation while sometimes decreasing transcription, and that it leads to increased acetylation at many peptides but decreased acetylation at others, it doesn't really uncover how. The consequences of Romidepsin treatment are presented in a more list-like fashion than with a mechanistic narrative to understand them. In the present form, I think the study would make more sense for a different journal, but if one or more of the above issues can be more meaningfully addressed it would make it more suited for this journal. I do think the work makes an important contribution, and would be happy to read a revised version.

We appreciate your comments and helpful suggestions which have greatly improved our manuscript and the impact of our work. HDACi have broad and complex effects on protein acetylation and transcription. We more clearly point out in the revision that romidepsin counters a genome-wide deacetylation response, and we performed new analyses to investigate the relationship between histone acetylation and gene expression. Please see our response to Reviewer 1's concerns that you similarly shared.

SUGGESTIONS TO AUTHORS

© 2026. Published by The Company of Biologists under the terms of the Creative Commons Attribution License (https://creativecommons.org/licenses/by/4.0/).

Essential revisions and problems that need to be addressed:

1. The A485 studies are not well controlled. Given that a theme of the paper is that pharmacological inhibitors can have broader effects than intended, it stands out that neither efficacy nor specificity of A485 is demonstrated here. I think the most straightforward way to address this would be to treat larvae with A485, Romidepsin, or DMSO, and use mass spec to assay the acetylation level of CREBBP peptides, as the intended target of A485, as well as at least a couple other proteins that are strongly differentially acetylated by Romidepsin but not expected to be targets of A485. Loss of acetylation of CREBBP but not other Romidepsin-senisitve peptides would show both efficacy and specificity. Another strategy would be to use a different, orthogonal pharmacological inhibitor of CREBBP (if available) and show the same effects. Even better would be a mutant version of CREBBP that could act as a dominant negative (missing key acetylation residues or even a catalytically impaired mutant, as in PMID: 29551561), but I'm not sure if that is feasible in this axolotl regeneration context.

Researchers have been publishing HDAC inhibitor papers for over 10 years without showing how the inhibitors affect specific histone and non-histone acetylation changes; in other words, without showing efficacy or specificity. Although there have been fewer studies, A-485 is well-established to block the acetyltransferase domain of Crebbp/Ep300 and thus the ability of these two proteins to acetylate thousands of target proteins, including the possibility of autoacetylation which you advance as a possible mechanism. While we would like to perform MS/MS after A-485 inhibition, to identify Crebbp/Ep300 targets, we do not have funding to perform such an analysis. In the discussion, we addressed the need to functionally test specific protein acetylation sites in future studies.

2. The read depth for ChIP-SEQ data needs context, since the axolotl genome is huge. 11-30 million reads may not be deep enough for reproducible peak calling for these very abundant marks. (For the human genome, one would typically shoot for about 30 million reads for these marks, and that genome is 1/10th the axolotl's size).

We were initially concerned about this too, but there are a couple of important factors to consider. The most important is that genome size does not scale with organismal and genome complexity. The axolotl has about the same number of genes as human, and a roughly similar developmental biology (more or less), so we might expect that the numbers of regulatory elements controlling expression of these genes would be similar. In our study, we report a total of 133 Mb assigned to H3K9ac peaks for intact tail, which is comparable to the summed lengths 138.7 Mb of H3K9ac peaks across the human genome (cell culture). Even the union of peaks from all samples in our study: 235 Mb (H3K9ac) and 186 Mb (H3K27ac), is comparable to human DMSO 138.7 Mb (H3K9ac) and 168 Mb (H3K27ac). (PMID: 29240919).

The number of sequenced fragments in our study (100-150 million fragments per sample) is comparable to (higher than) numbers reported from the only published study of genome-wide chromatin profiling of an axolotl genome, which used a Cut&Tag - "ChIP-seq like" sequencing approach (PMID: 38788714). In that published study number of sequenced fragments per sample ranged from 19 to 265 million (M), with average numbers for H3K27me3 = 37M, H3K4me1 = 95M, and H3K4me3 = 69M.

Our analyses also suggest that we approached sampling saturation for the sampleable peaks:

1) As shown in Supplementary Table 1, the number of reads sequenced for each sample was 100-150 million, but for our samples 50% or more were filtered out as duplicates (alignments starting at the same nucleotide) or multimappers. Therefore, deeper sequencing, while more expensive, would not be expected to further benefit the study.

2) The quality of peaks can also be measured using FriP-scores, which we now report for all samples. Our Frip-score values (H3K9ac: 0HPA 0.224, C-6HPA 0.127, R-6HPA 0.331; H3K27ac: 0HPA 0.133, C-6HPA 0.045, R-6HPA 0.22) exceed the ENCODE guidance threshold of >0.01 (PMCID: PMC3431496), despite the expected complexity associated with measuring these in a developmental context and under chemical perturbation.

© 2026. Published by The Company of Biologists under the terms of the Creative Commons Attribution License (https://creativecommons.org/licenses/by/4.0/).

I think it's okay to create a union set, but what is the level of concordance in peak calls between replicates? That will give a sense of whether the sequencing is deep enough/well-powered enough. Some Venn diagrams and statistical analysis would help here.

The union set is essential for making apples to apples comparisons between samples. Due to the absence of a defined set of peak regions from an external source (e.g. Encode peaks are abundantly available for humans, we do not have this for axolotl), it is necessary to define a set of regions that can be directly compared through some method. Generating a union allows us to give equal weight to all experiments and prevents us from missing or under sampling peaks that aren't present in the control.

Owing to the need to use exceedingly large pools of tissue samples and preparative costs, we did not examine replicate samples within our parallel ChIP-SEQ and CUT&RUN experiments. However, we note that pooling and replication both serve to reduce individual-specific noise, and we did replicate/validate our results by repeating experiments using different biological replicates and technical approaches.

Also, could the authors please comment further in the methods on how data were normalized to ensure that fewer/more peaks weren't just called because of read depth—were libraries downsampled to a uniform number of reads and compared, for example? Normalized by FPKM or similar? I know these are fairly standard processing steps but it would be helpful to be able to see how they were applied.

In the revised manuscript, we include additional methodological details. Narrow peaks were called by MACS2 individually for each sample based on all uniquely mapped and filtered reads, without any subsampling. MACS2 calls peaks based on the coverage depth differences across a reference sequence, and relative to input, chromatin scaling coverages internally.
To compare acetylation signal between samples, read depth was normalized as described in the methods "genome-wide read coverage was computed and normalized by scaling to 1 million mapped reads with BEDTools v2.27.1 and bedGraphToBigWig". We now clarify that this corresponds to the standard reads per million (RPM) terminology.

Your concern motivated us to determine if down sampling would yield a different signal. We calculated global breadth and mass changes in H3K9ac from BAM files that were down sampled to the number of reads in the C6-HPA file and observed the same trend: acetylation decreased in C-6HPA compared to 0HPA and increased for R-6HPA compared to C-6HPA. Since this approach does not use all data, we prefer the normalization methods that we used.

3. The discordance between increased acetylation and increased transcription is very interesting, because it unseats our general expectation of how transcriptional regulation works. To me, there are two ways one should test the relationship between acetylation and transcription after Romidepsin. One is to ask if transcriptionally upregulated genes also have increased acetylation. This the authors looked at (at least for 29 genes), and seems to be generally but not always true (Supp Fig 3). But it's at least equally important to ask the question the other way, which I don't think was done: do genes with increased acetylation (in promoters or gene bodies) have increased transcription? So when the authors say "Gene ontology (GO) enrichment analyses of genes associated with romidepsin-sensitive promoters (red dots in Figure 3) identified a variety of enriched developmental processes and cell signaling pathways, including TGF, WNT, BMP, NOTCH, EGFR, PDGF, VGF, and ERBB (Supplementary Table 2)"—are these genes transcriptionally increased, decreased, or unchanged relative to DMSO treatment at the same timepoints?

Please see our response to Reviewer 1 comment 1. We performed new analyses to examine the relationship between histone acetylation and gene expression. We think these analyses greatly improve our manuscript and the impact of our work.

4. I didn't really follow what the authors make of the decreases in acetylation that are observed for non-histones like the clotting factors after Romidepsin treatment. What hypotheses may explain these observations? More specifically, I think the mass spec measurements would allow them to parse a loss of protein abundance (all peptides for that protein decrease, explainable by decreased transcription or translation, or increased degradation) versus a loss of acetylation (the ratio of

© 2026. Published by The Company of Biologists under the terms of the Creative Commons Attribution License (https://creativecommons.org/licenses/by/4.0/).

acetylated to non-acetylated peptides for that protein decreased, explainable by loss of a non-histone acetyltransferase or gain of a deacetylase). For which of the non-histone proteins that have decreased acetylated peptides are they seeing a loss of protein, and for which are they seeing a loss of acetylation? Clarifying this would help the reader better understand whether these are changes that might be caused by decreases in transcription, such as by CREBBP. I think it would be helpful if some of the non-histone data appeared in a main figure, rather than just a supplemental table, which was a bit hard to parse.

Please see response to Reviewer 1 concerning acetylation stoichiometry. We observed an increase in acetylation for hemostasis proteins, not a decrease. In the revised manuscript, we include additional writing in the discussion section to explain the significance of observing increased acetylation of hemostasis proteins at 6 HPA.

5. Romidepsin treatment was previously noted as remarkable by this group because it led to altered regeneration outcomes after just 1 minute of treatment, which they stress again here. But the effects assayed are all after 6 or 24h. Does Romidepsin treatment for 1 minute change acetylation of any of these targets? Maybe the authors could assay a few of the marks in Figure 1 that were the strongest responders after 6h to see if those also respond to a 1 minute treatment. If they don't respond, then the effects of Romidepsin on regeneration may have more to do with clotting proteins and immediate wound response than transcription?

It is an interesting idea to further explore the effect of romidepsin dosage on histone acetylation (Figure 1). However, if no change in histone acetylation is observed after a 1 minute romidepsin treatment, this wouldn't rule out transcriptional regulation by non-histone proteins. It would be necessary to perform both MS/MS analysis of histone PTMs and non-histone proteins to investigate this further. We do not have funds currently available to explore the effects of 1-minute romidepsin treatment.

Minor comments and suggestions:

1. Standard capitalization conventions should be followed: CUT&RUN, ChIP-SEQ

Done

2. I think a schematic Figure 1 that laid out how the experiment was done and how the data were analyzed might make the overall workflow and types of conclusions drawn easier to follow.

Done

3. The parallels drawn between regeneration and cancer in the Introduction (and a bit elsewhere, too) feel a little too broad or forced. Tumor suppressors and oncogenes have specific definitions. Not every inflammation-promoting factor should be equated to a tumor suppressor.

We deleted sentences in the introduction that equated tumor suppressors and oncogenes between axolotl regeneration and cancer.

3. Could the authors add a bit more comment about whether/how some of their observations may be influenced by tissue- or cell type-specific effects? For example, could gains of acetylation in one cell type cause a local increase in transcription that's being swamped out by relatively low abundance of that cell type, or losses of transcription in a different cell type?

We comment a bit further in the revision when discussing this caveat. We appreciate that ongoing efforts to further develop single cell epigenetics approaches should eventually allow us to specifically measure site-specific changes in histone modification in individual cell types within complex tissues.

4. It is just generally always hard to keep manuscripts about lists of genes, directionality of regulation, and lists of GO terms clear and easy to follow. Can't be prevented. I do think the authors could help their readers out by going over the Results and Discussion once more. It would help to break paragraphs into smaller ideas with clear topic sentences and clear conclusion

© 2026. Published by The Company of Biologists under the terms of the Creative Commons Attribution License (https://creativecommons.org/licenses/by/4.0/).

sentences, and make sure that the logic in each sentence and paragraph follows easily from observation to conclusion. (Sorry for this—I have written plenty of mushy paragraphs about GO terms myself, and I know it is challenging to fix).

In the revised manuscript, we reworded sections of the results and rewrote the discussion for clarity. We worked hard to make the revision a more logical and impactful read.

Transfer to Biology Open

First decision letter

MS ID#: bio.062726

MS Title: Histone and Non-Histone Protein Acetylation in a Tail Regeneration Model (Ambystoma mexicanum)

Authors: Nataliya Timoshevskaya; Raissa Cecil; James Schwartz; Jeramiah Smith; Stephen Randal Voss

I am happy to tell you that your manuscript has been accepted for publication in Biology Open, pending our standard publication integrity checks. It was accepted on 5th June 2026.

© 2026. Published by The Company of Biologists under the terms of the Creative Commons Attribution License (https://creativecommons.org/licenses/by/4.0/).
